# Supplementary figures and images for: Deep and continuous sedation until death in the French overseas departments
Source: PLoS One. 2025 Dec 5;20(12):e0337969. doi: 10.1371/journal.pone.0337969 (PMC12680175; doi:10.1371/journal.pone.0337969)

Supporting material 2: questionnaire sent to physicians


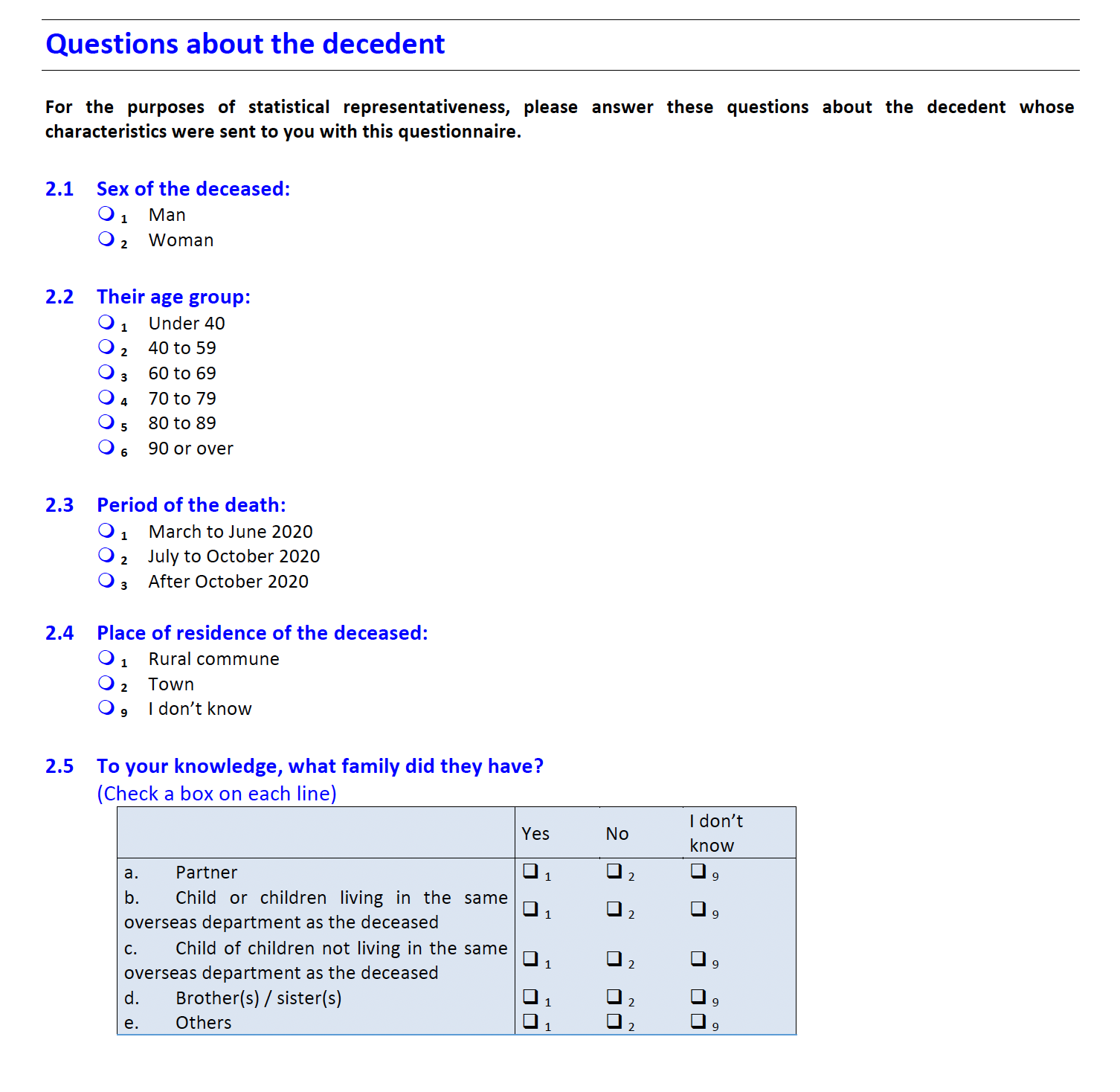


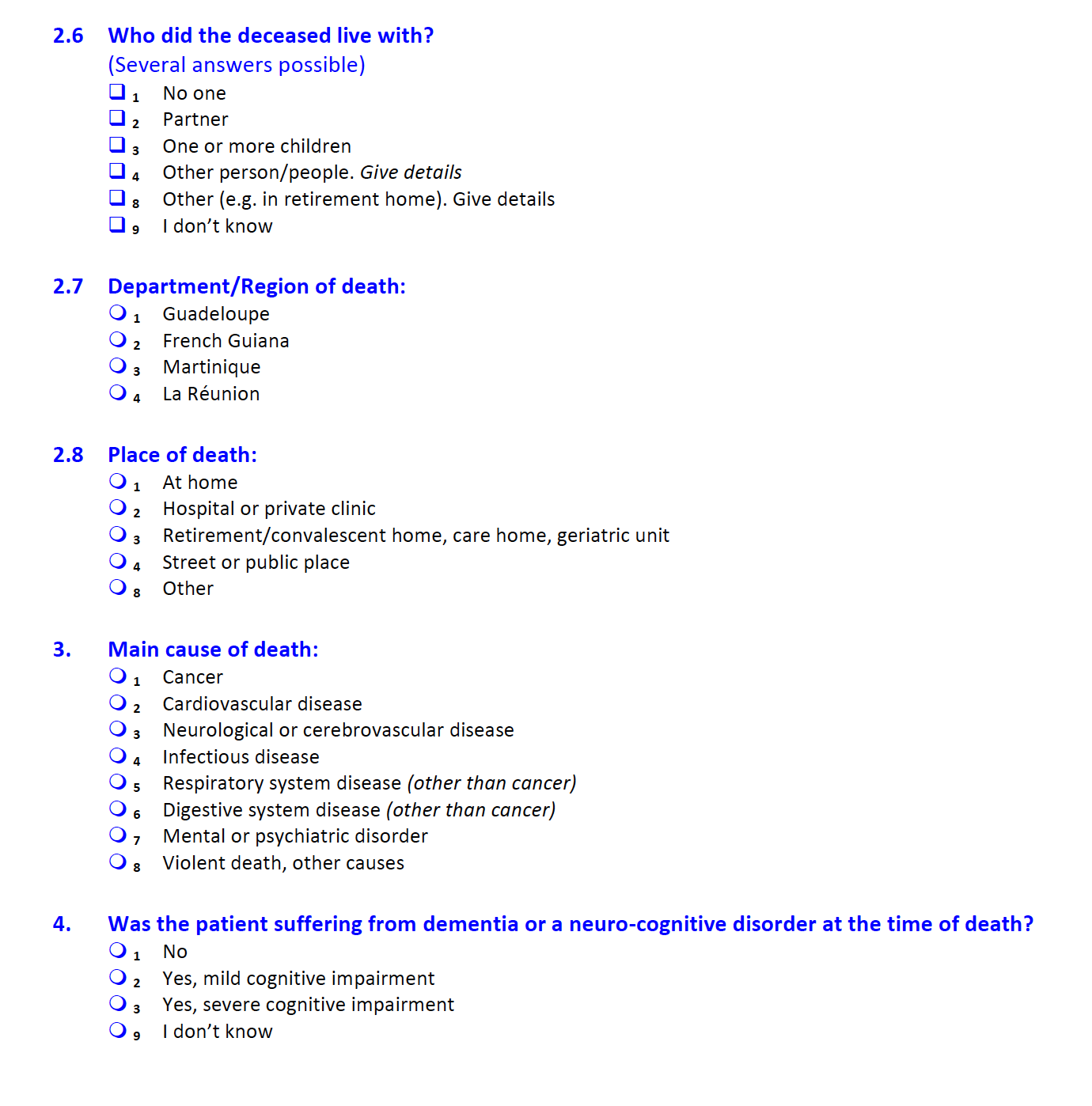


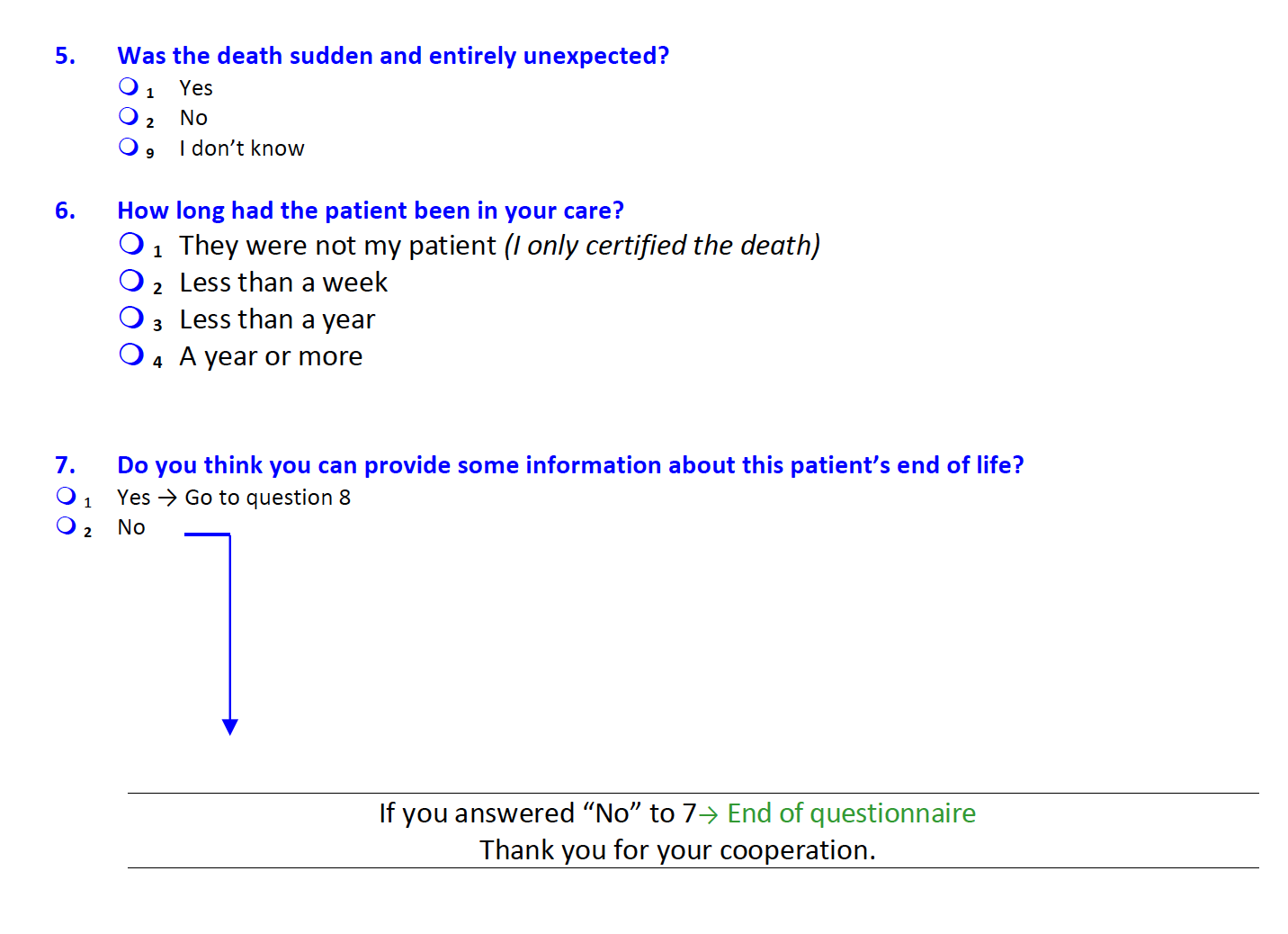


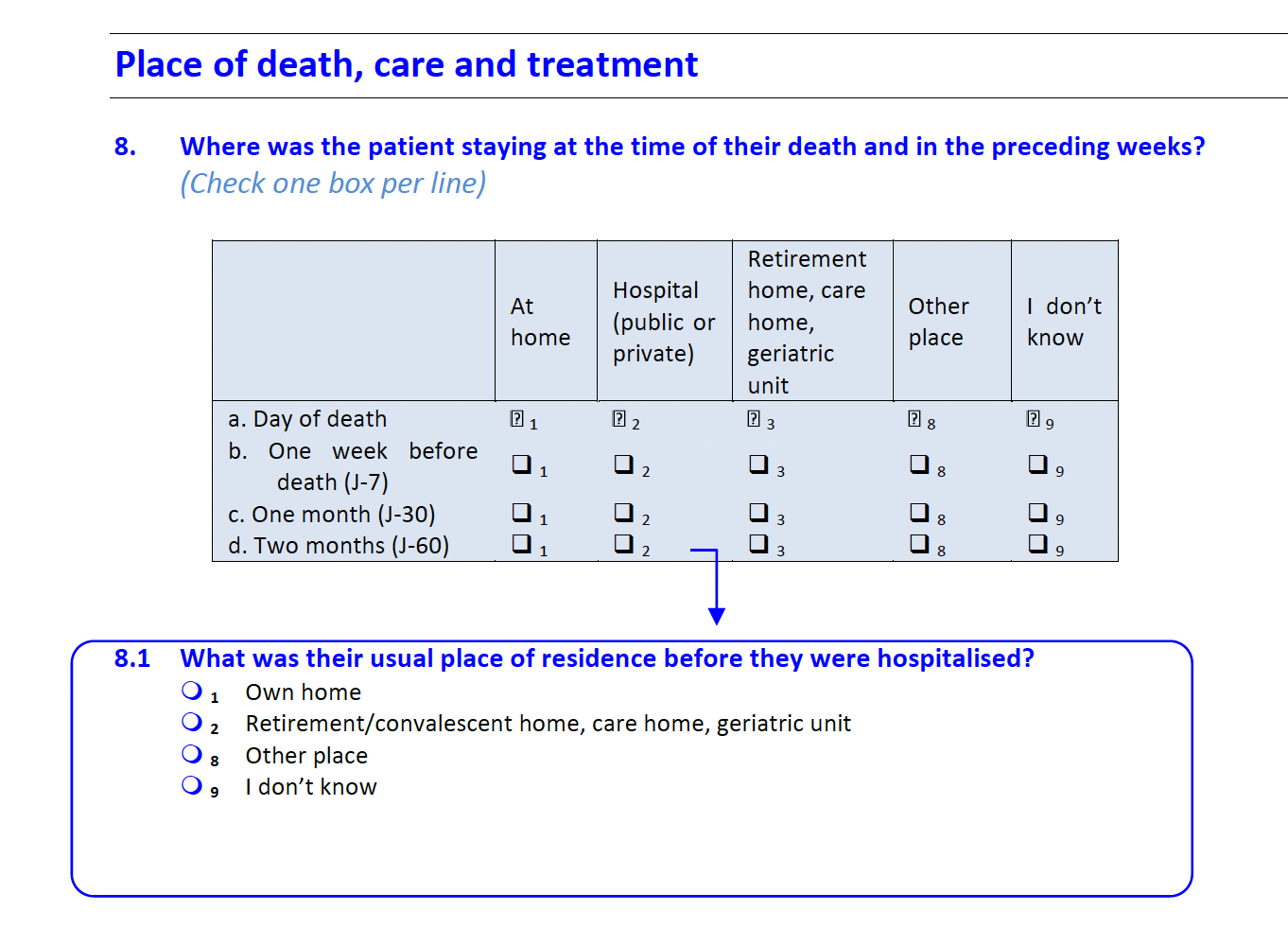


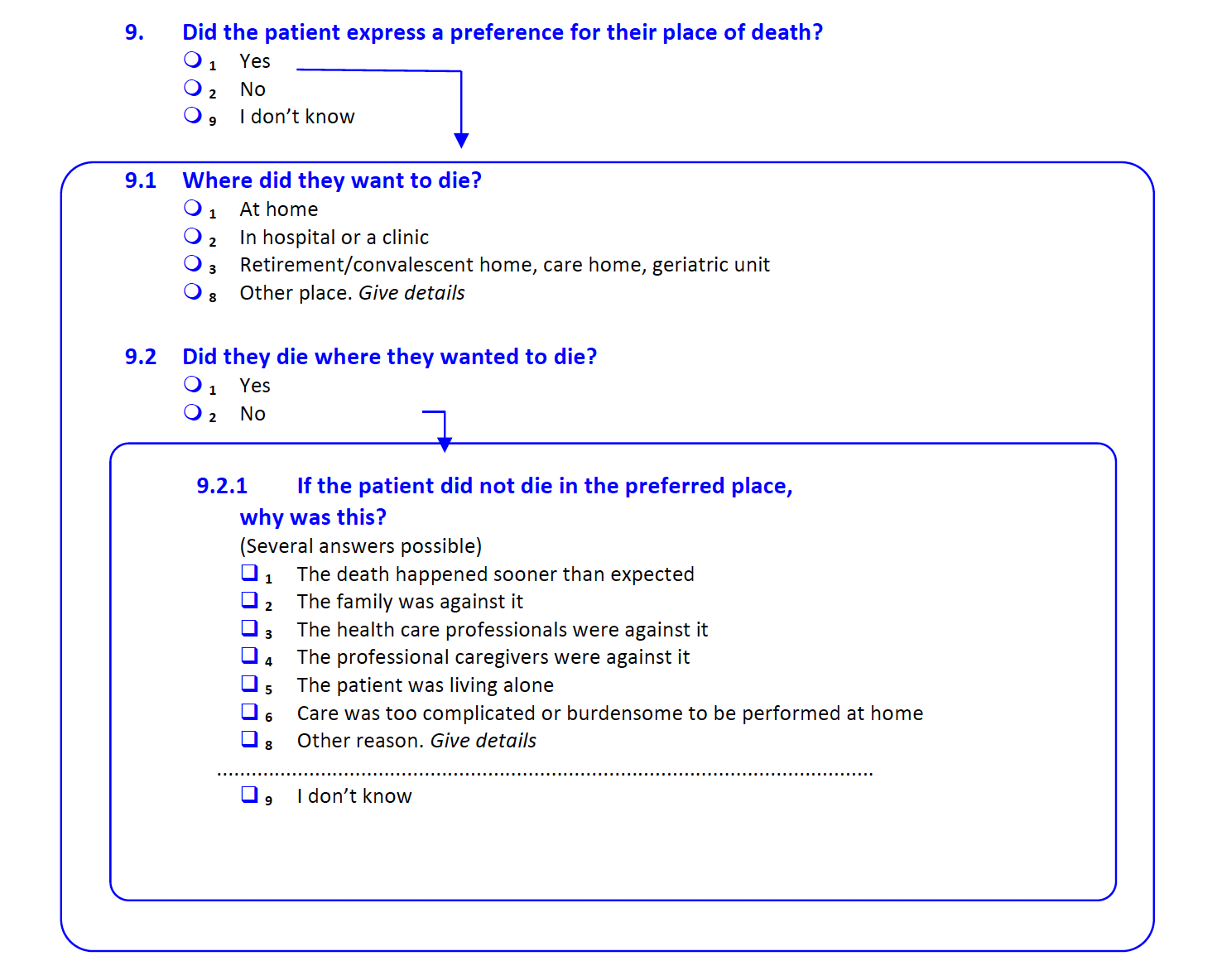


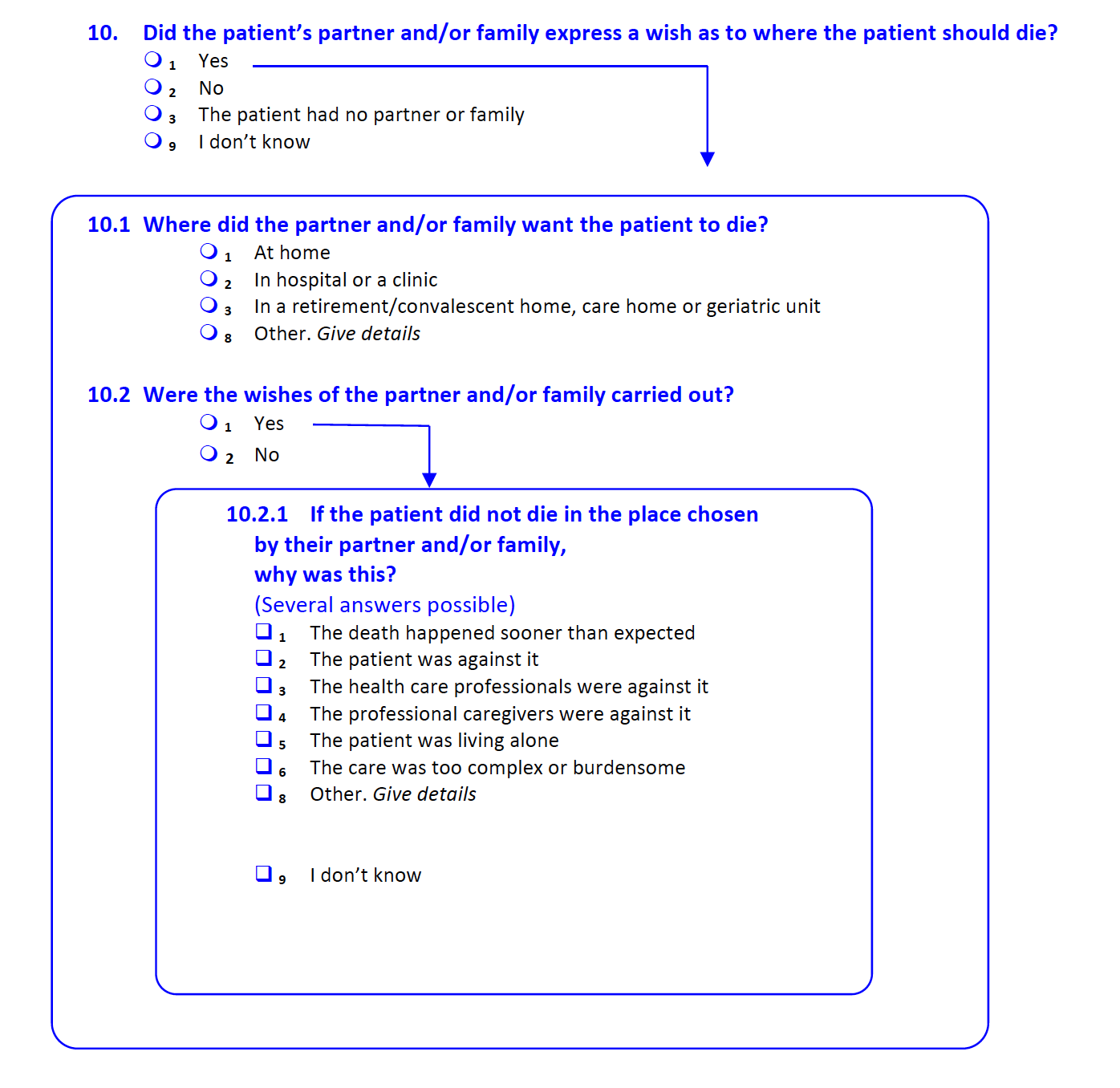


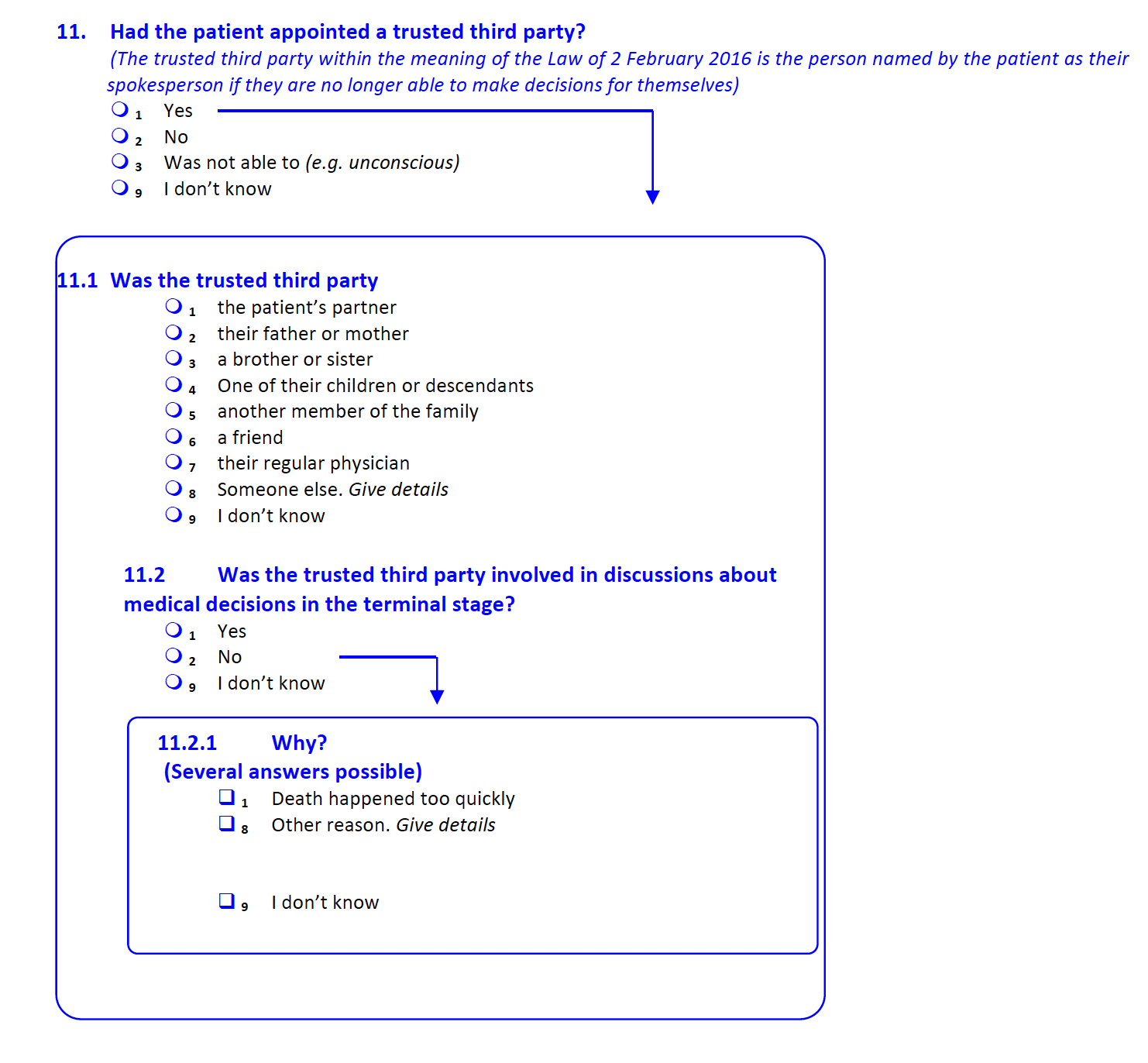


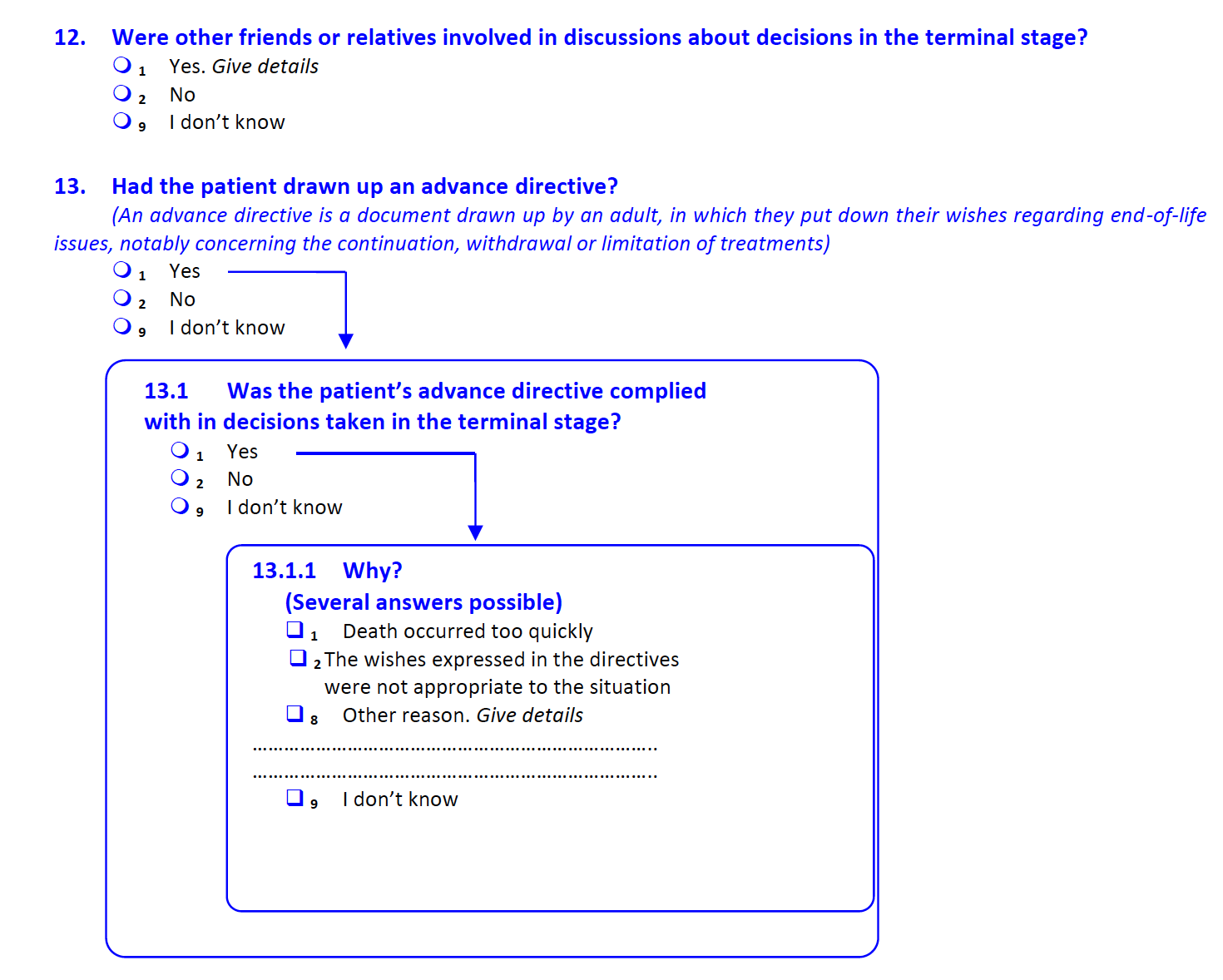


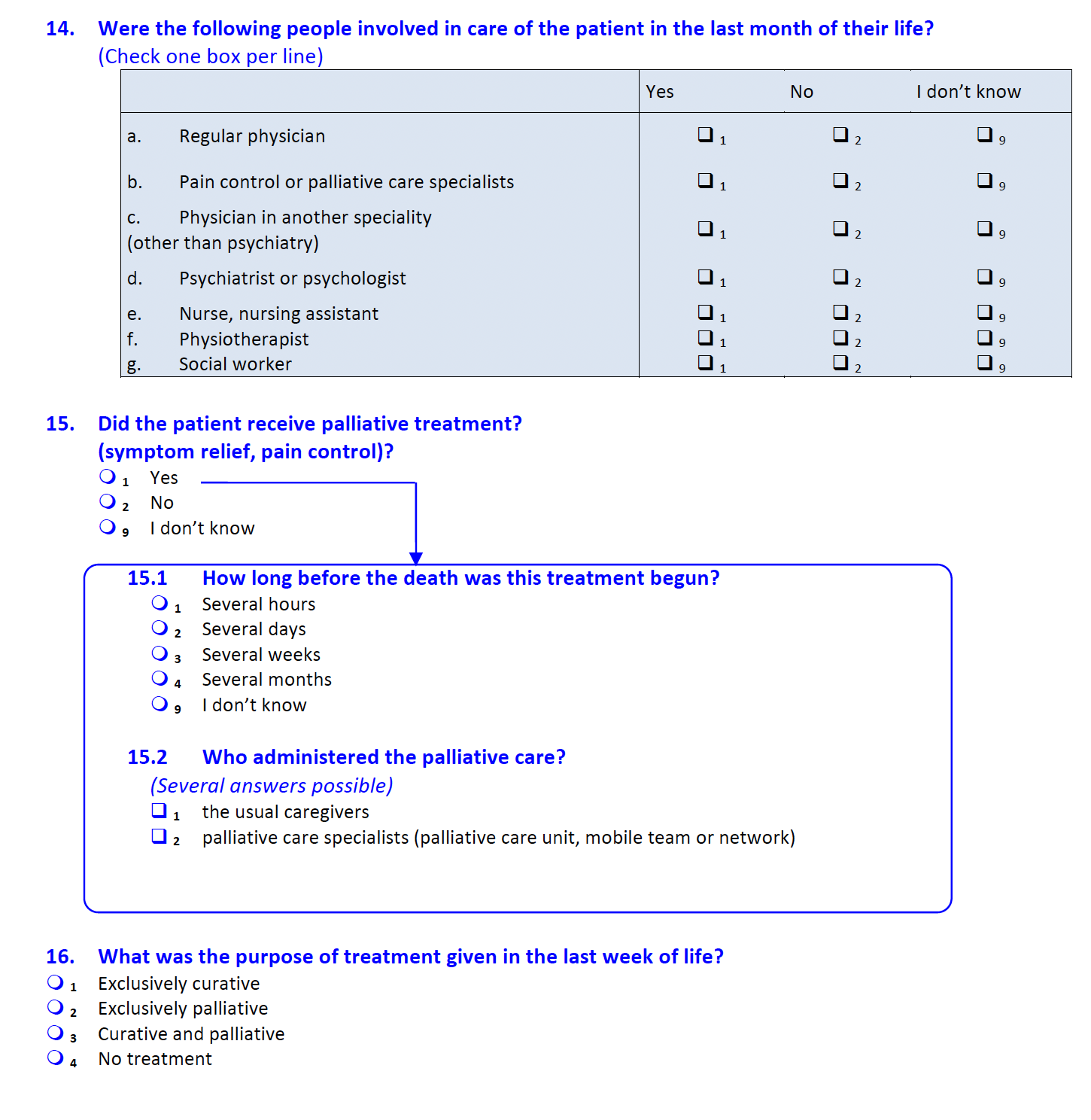


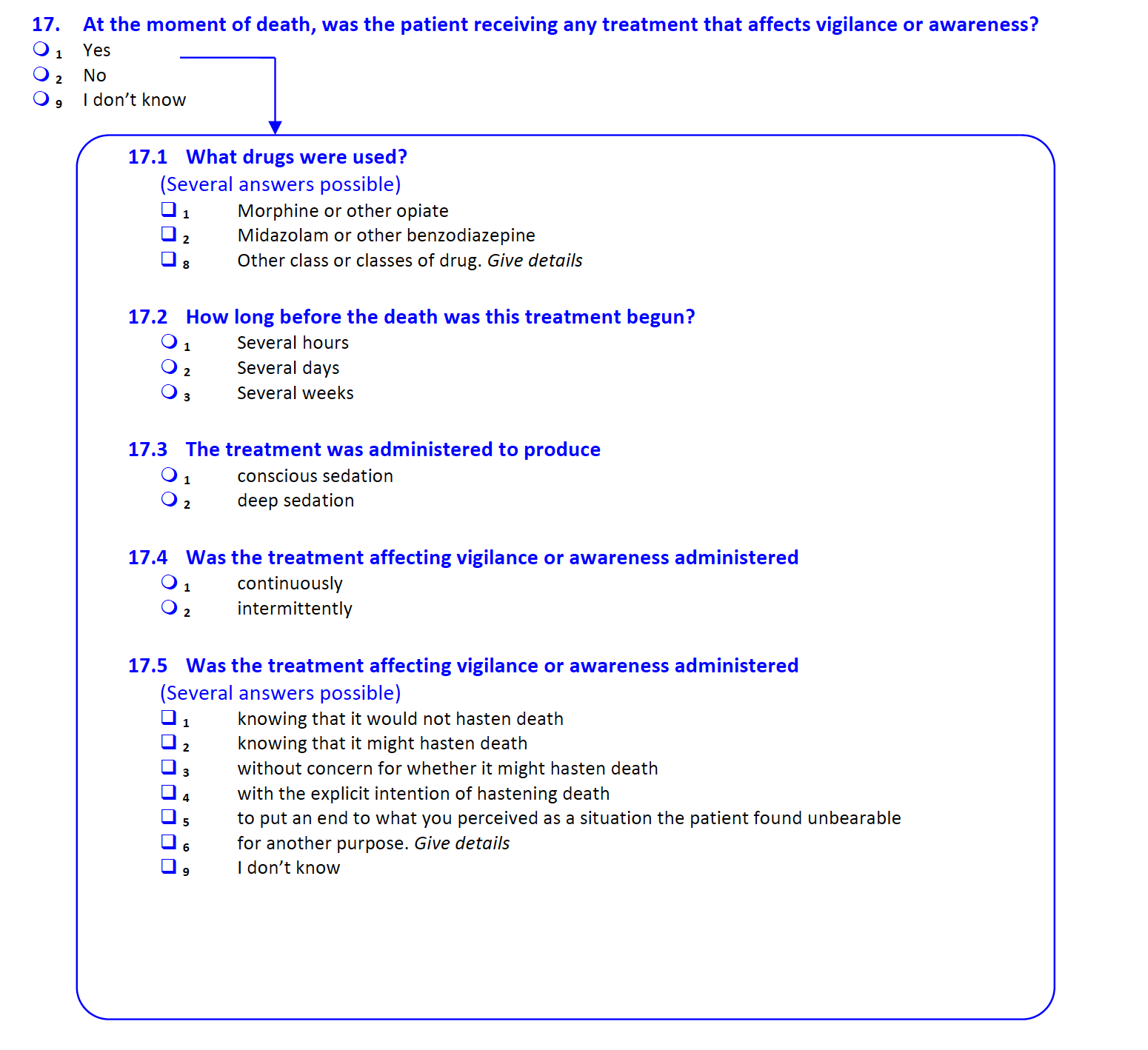


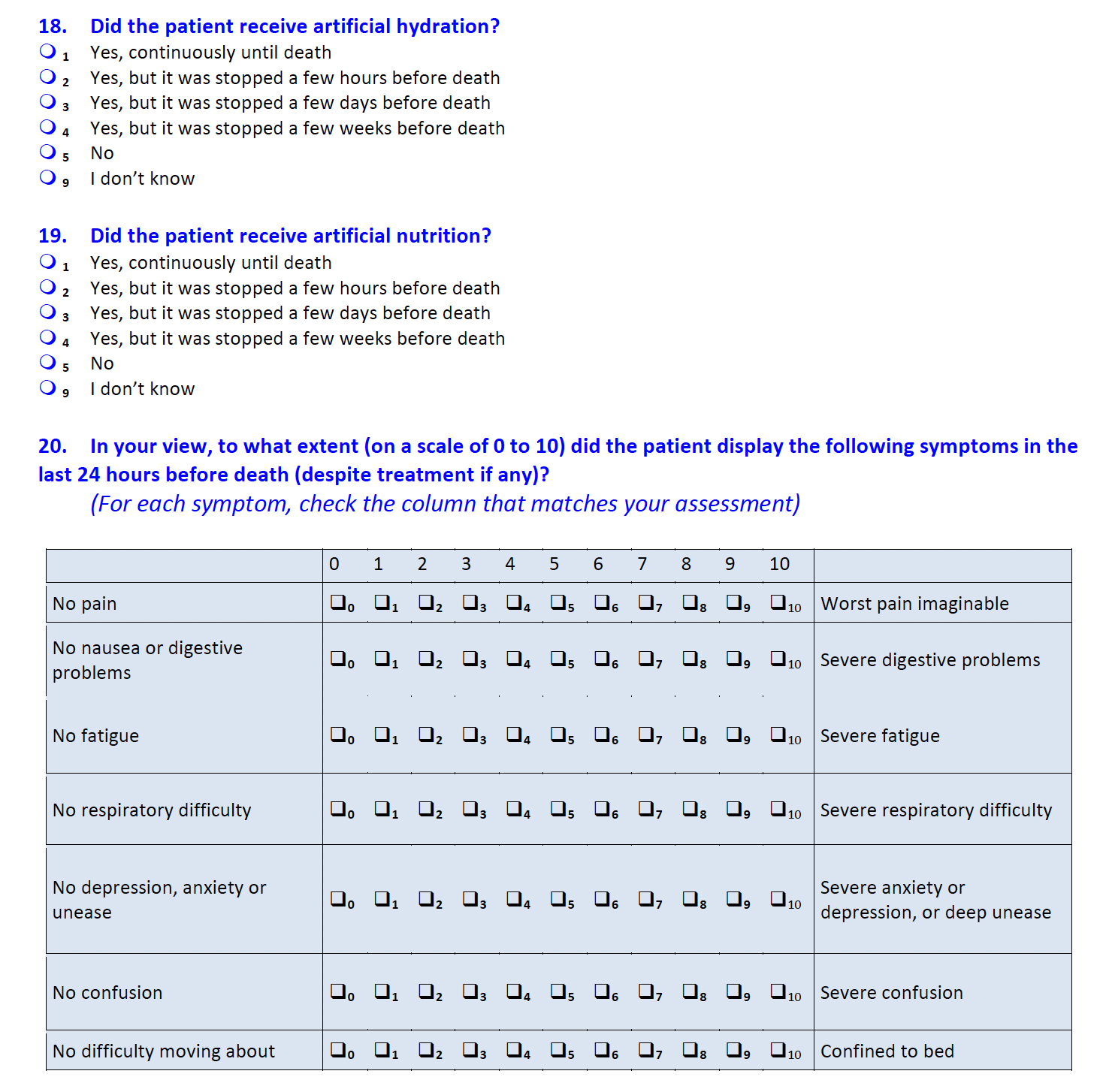


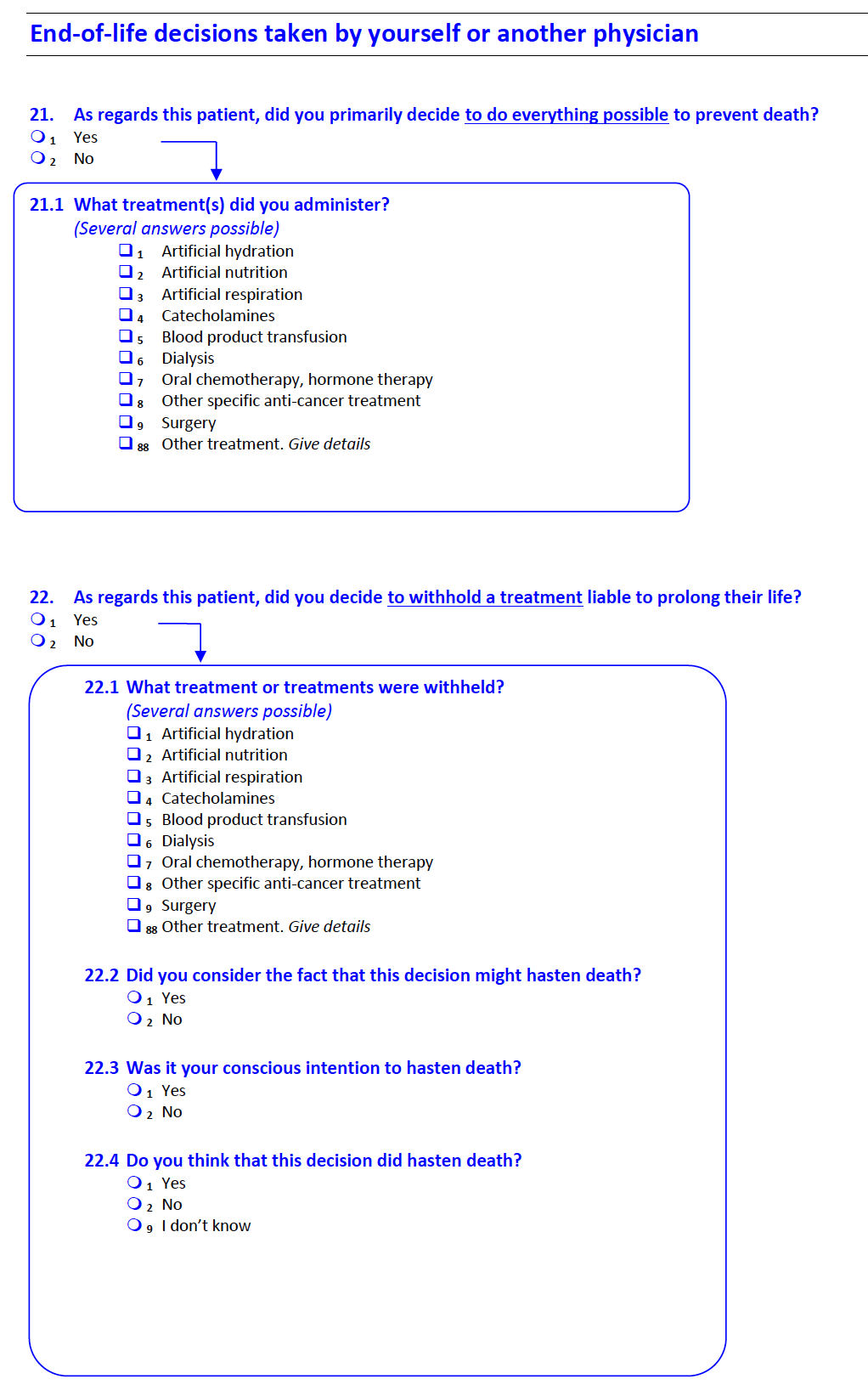


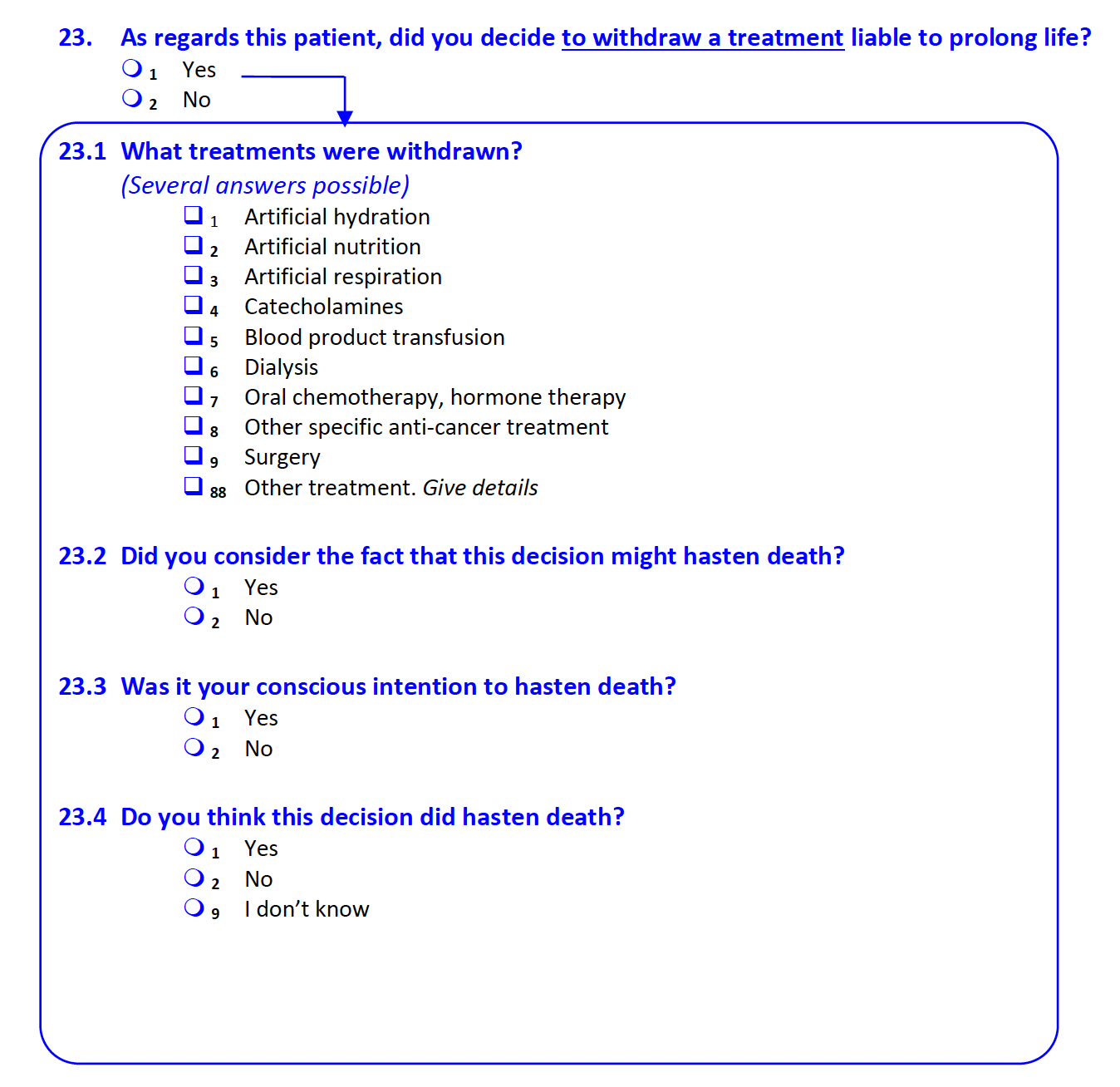


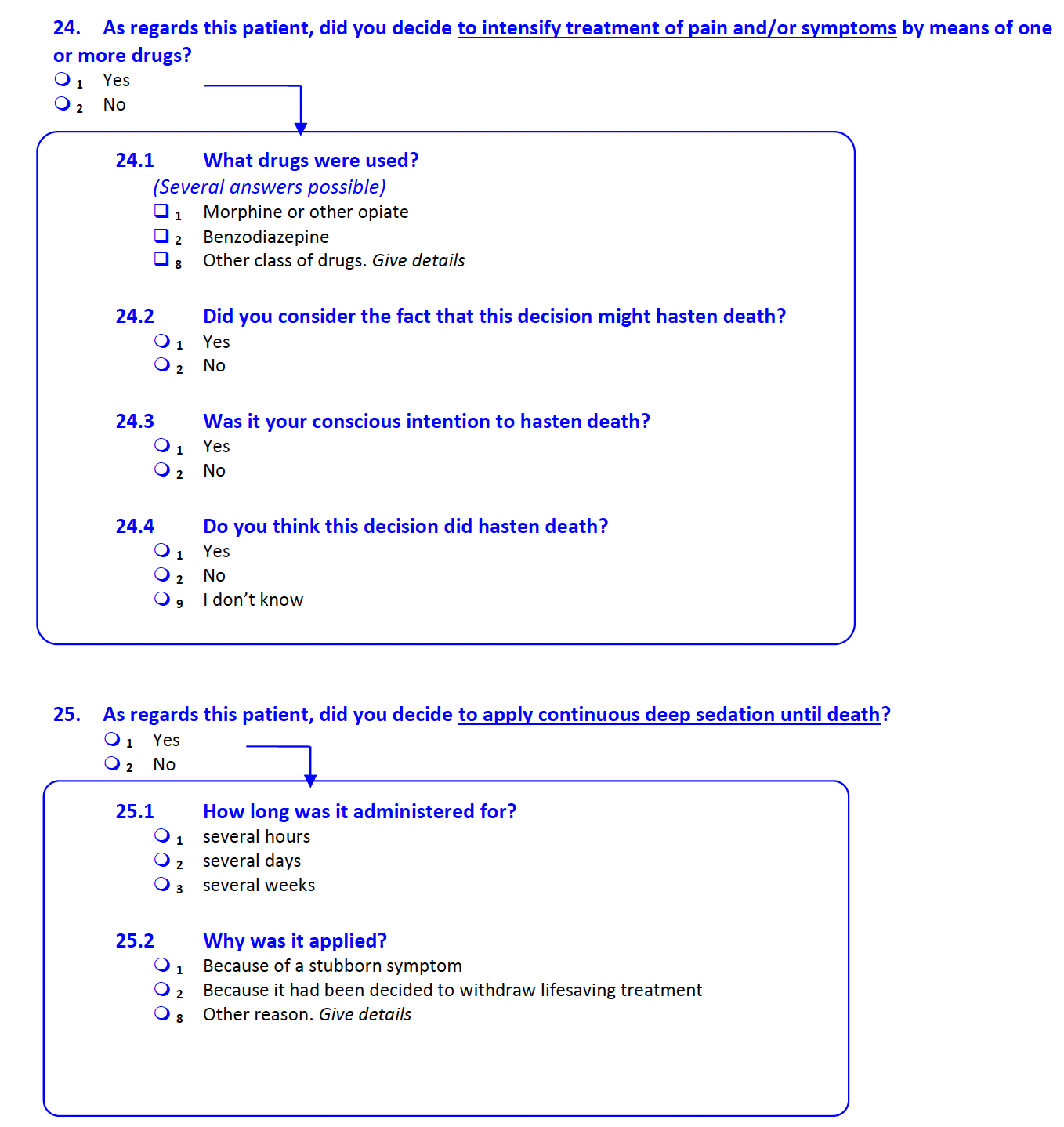


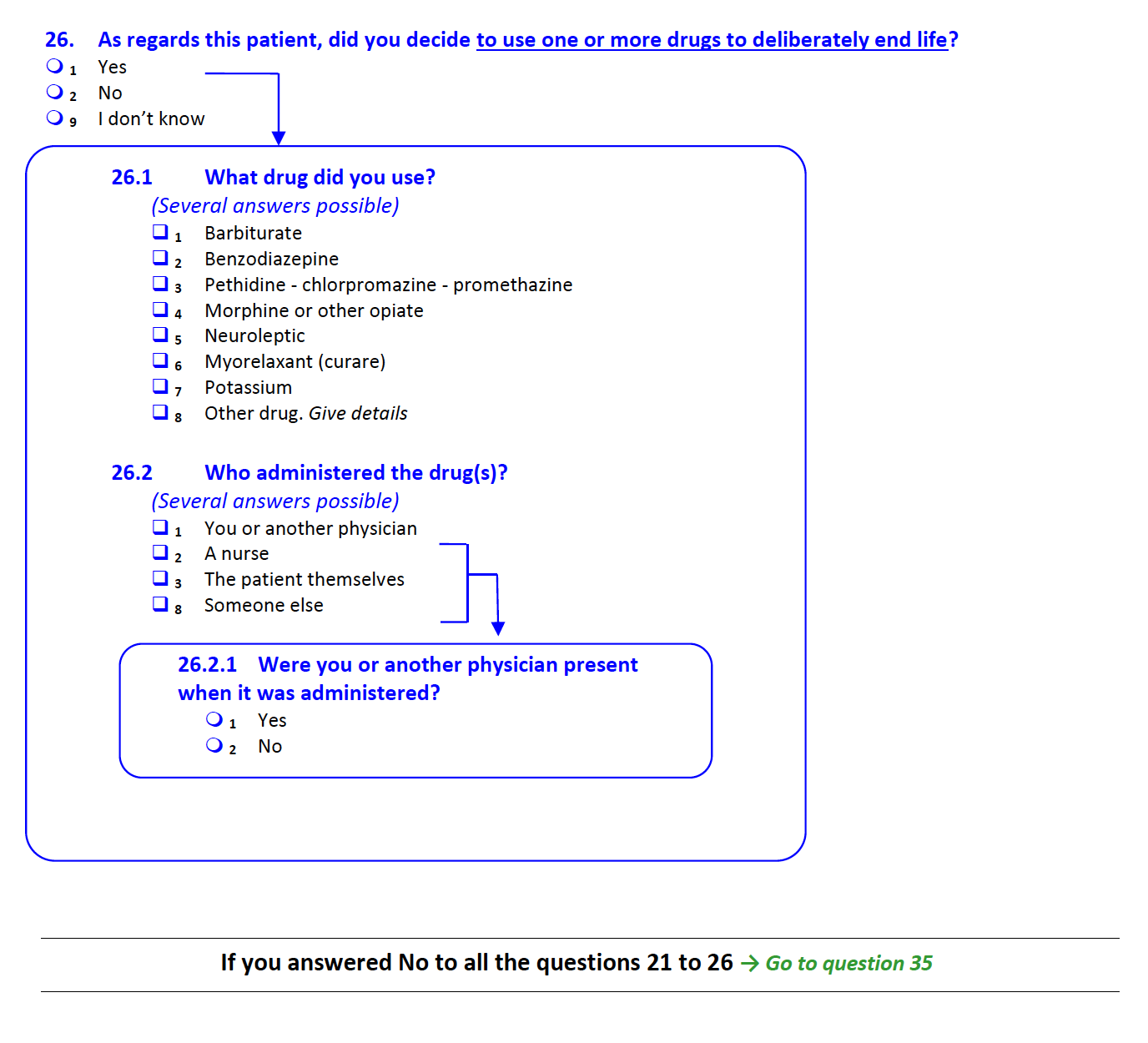


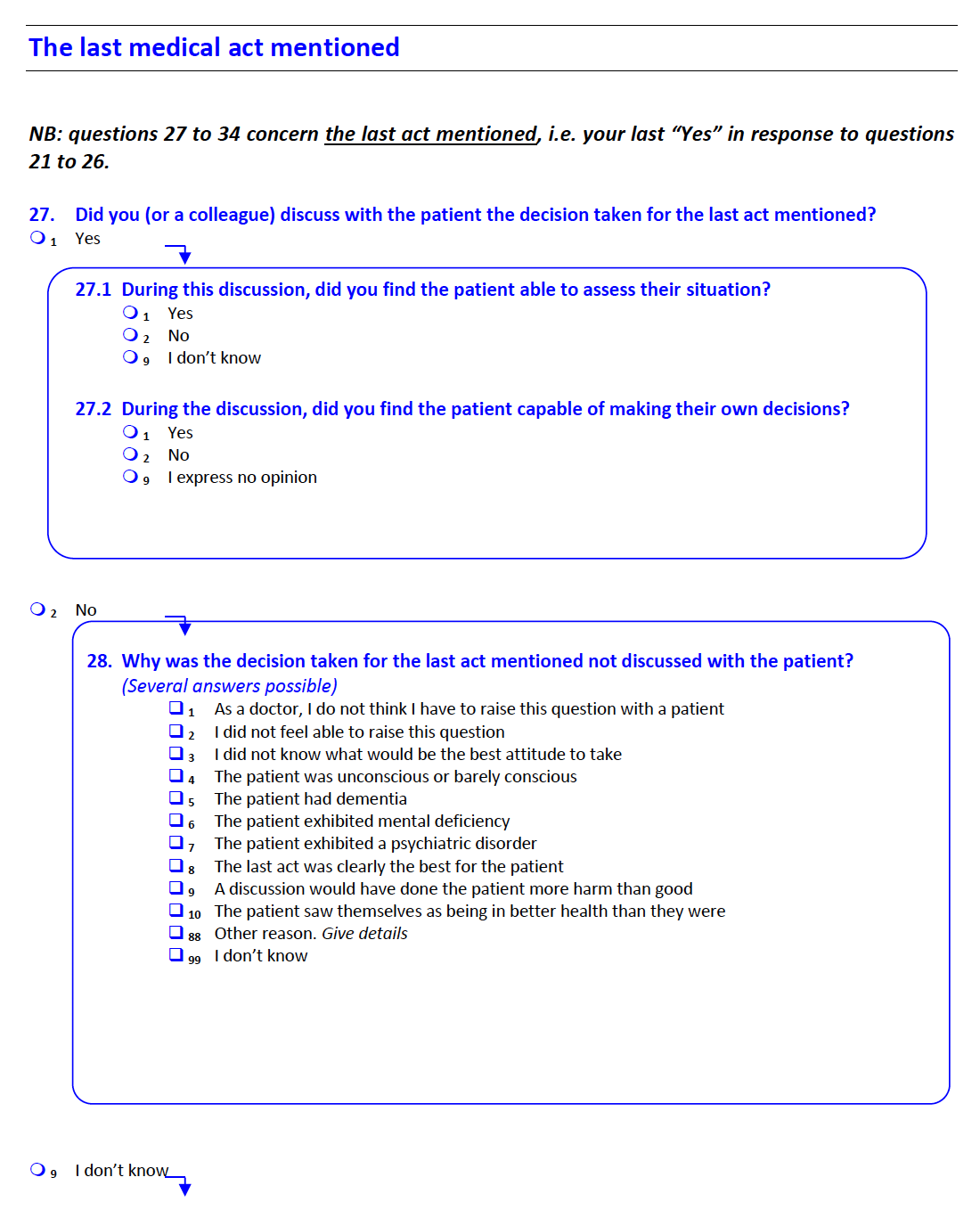


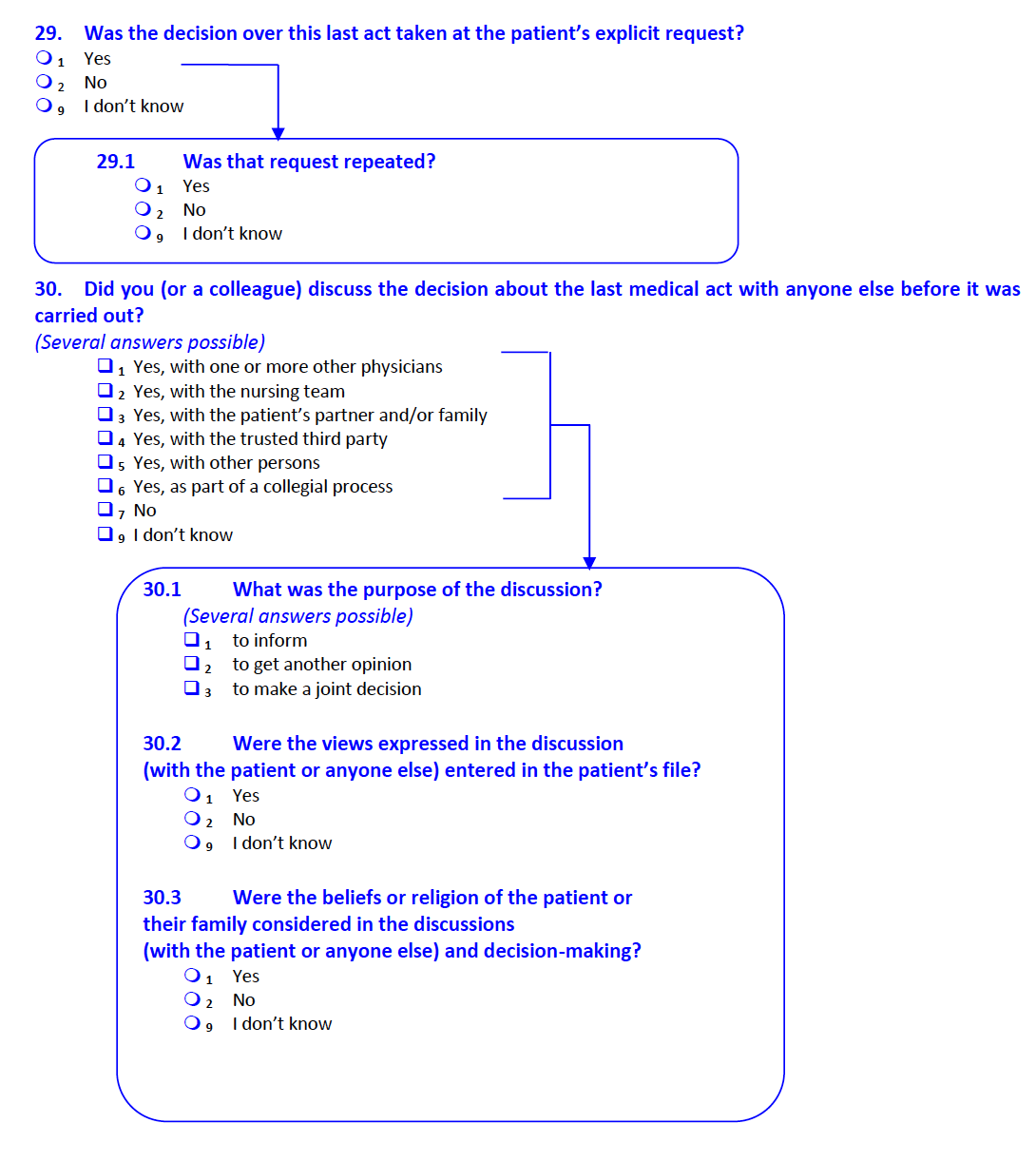


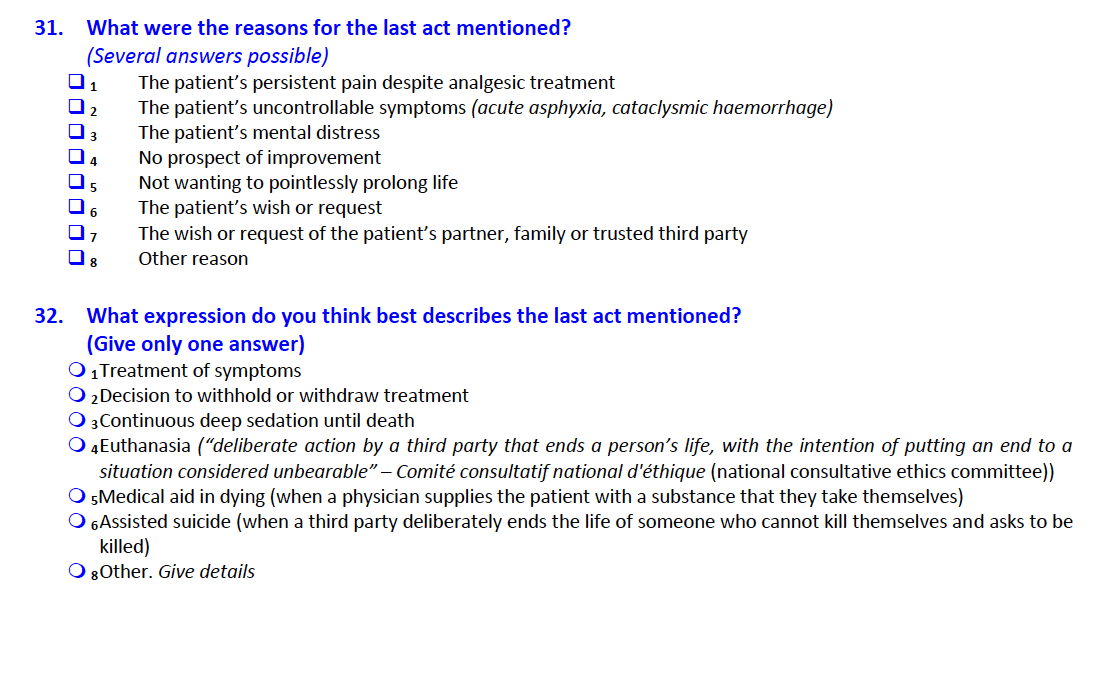


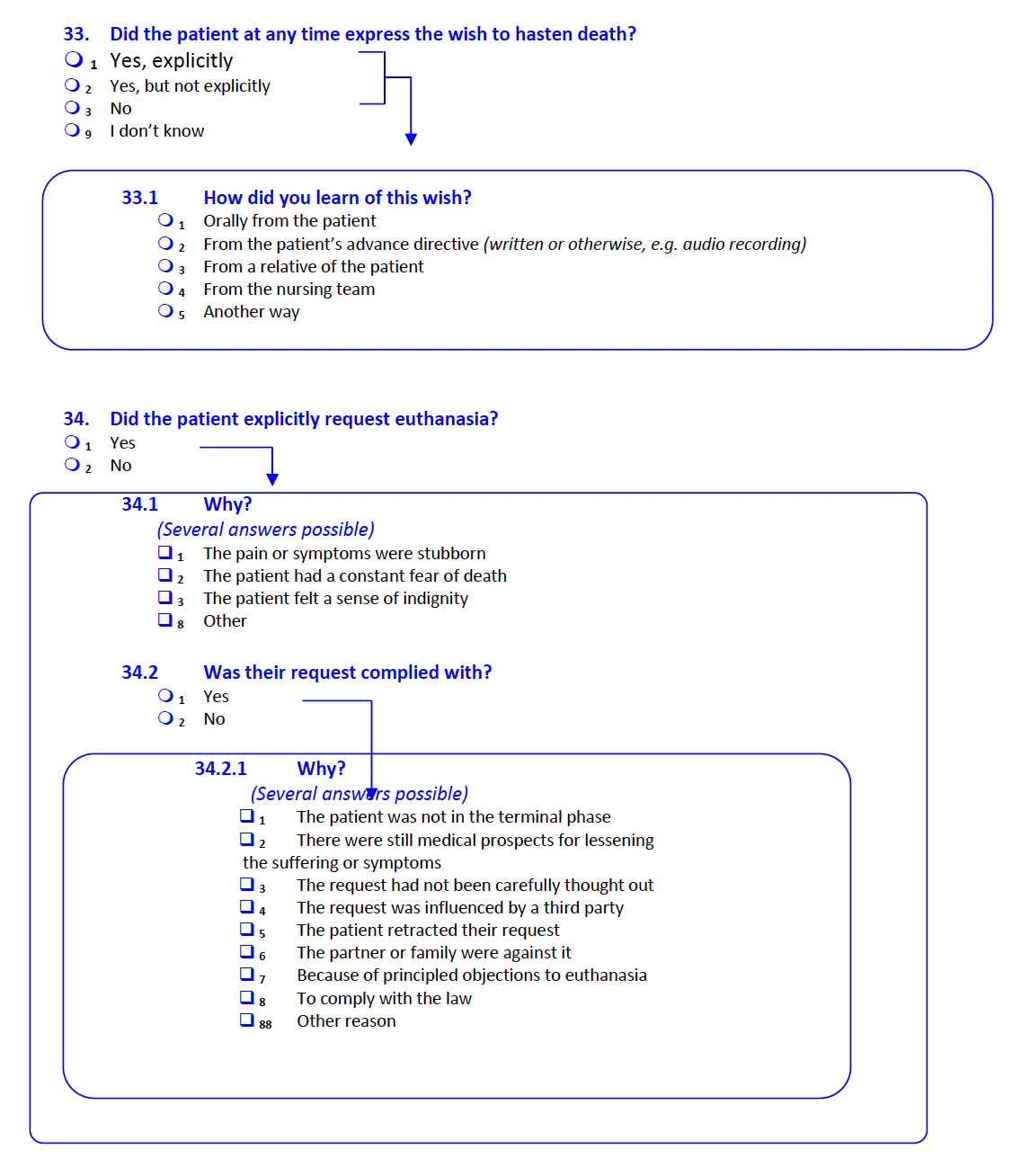


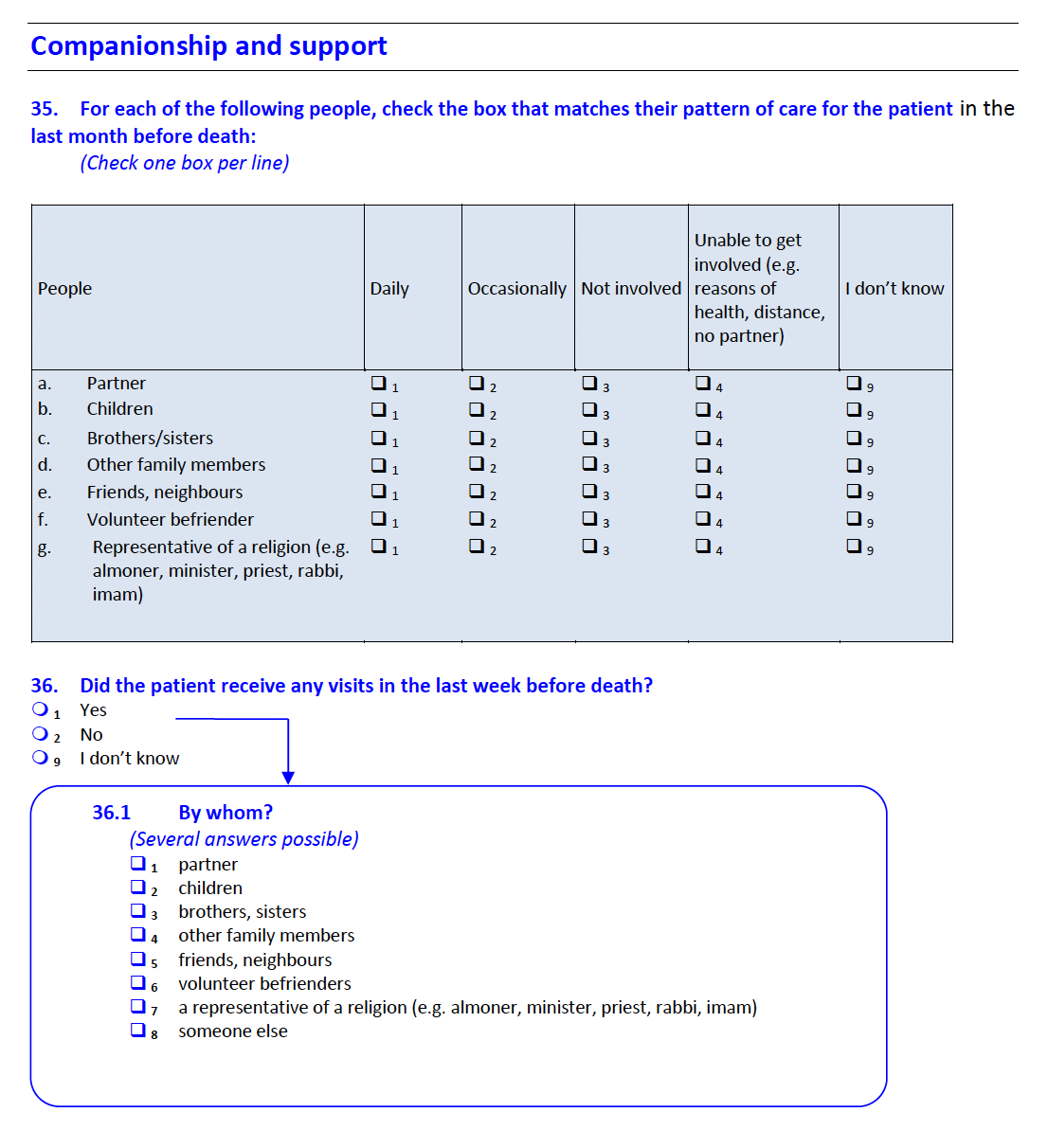


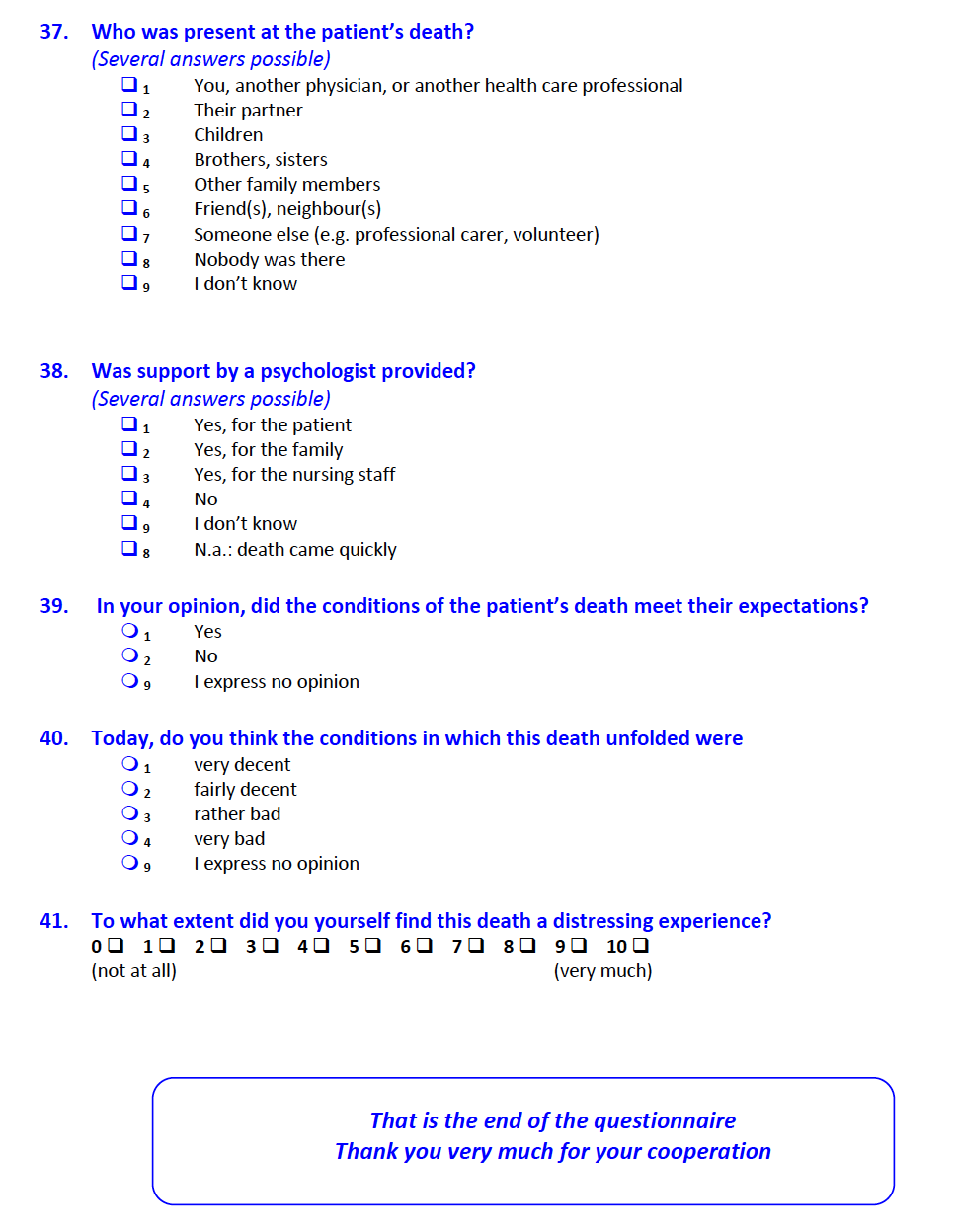

Supplement: S2 File — (DOCX) [file pone.0337969.s002.docx]
