# Supplementary material for: Deep and continuous sedation until death in the French overseas departments
Source: PLoS One. 2025 Dec 5;20(12):e0337969. doi: 10.1371/journal.pone.0337969 (PMC12680175; doi:10.1371/journal.pone.0337969)
Supplement: S3 File — (DOCX) [file pone.0337969.s003.docx]

Supporting material 3: search equations

- **Equation A**

Answer Yes to question 25 + Answer Yes to question 32=> Total n =62

Answer Yes to question 25 + Answer Yes to question 32+ Answer Yes to question 17 => Toal n= 58

Answer Yes to question 25 + Answer Yes to question 32+ Answer Yes to question 17 + Answer “Deep sedation” to question 17.3 => Total n = 40 certain CDSUD

*To simplify, we will now write as follows : 25 (yes) + 32 (yes) +17 (yes) + 17.3 (deep sedation) = 40* *certain CDSUD*

Then continued 17.3 equation A1:

25 (yes) + 32 (yes) +17 (yes) + 17.3 (vigilant sedation) = 19

25 (yes) + 32 (yes) +17 (yes) + 17.3 (vigilant sedation) **+17.1 (midazolam yes)** => 15 very likely CDSUD (physicians saying they did a vigil sedation with midazolam, but they put CDSUD for questions 25 and 32)

25 (yes) + 32 (yes) +17 (yes) + 17.3 (vigilant sedation) **+17.1 (midazolam no but morphine yes) =>** 4 very likely CDSUD => 1231 /1320 /1335 437 => (physicians saying they did a vigil sedation with midazolam, but they put CDSUD for questions 25 and 32)

Then continued 17.3 equation A2:

**25 (yes) + 32 (yes) +17 (yes) + 17.3 (empty)** = 1 very likely CDSUD : physician saying they did a vigil sedation with morphine, but they put CDSUD for questions 25 and 32

- **Equation B:**

25 (yes) + 32 (yes) => 62

25 (yes) + 32 (yes) **+ 17 (no) => 2 certain CDSUD** (physicians put no to questions 17 and 17.1, but in the comments, they put “cessation of life support” or “refractory symptoms” to justify deep sedation, which is therefore very likely to be CDSUD)

25 (yes) + 32 (yes) + 17 (empty) => 2 certain CDSUD => but they put 17.3 (deep sedation)

**Results equations A+B => 62 deep sedation which 42 probable CDSUD and 20 very likely CDSUD**

- **Equation C:**

25 (yes) => 151

25 (yes) +17 (yes) => 140

25 (yes) +17 (yes) +17.3 (deep sedation) = 71 (33 of which were unclassified were classified as very likely to be CDSUD and 38 classified as probable CDSUD)

Then continued 17.3 equation C1

**25 (yes) + 17 (yes) + 17.3 (vigil sedation)** = 66 (of which 19 are already classified as very likely to be CDSUD, leaving 47 unclassified)

25 (yes) + 17 (yes) + 17.3 (vigil sedation) + 32 (euthanasia) => 8: 1 classified as very likely CDSUD because they ticked CDSUD in question 32, and 7 classified as probable CDSUD

25 (yes) + 17 (yes) + 17.3 (vigil sedation) **+ 32 (empty)** => 5: 1 assisted suicide, 2 stop life-sustaining treatment classified as very likely to be CDSUD, 1 refractory symptom classified as very likely to be CDSUD, and 1 classified as uncertain because they put in comment “died of old age”

25 (yes) + 17 (yes) + 17.3 (vigil sedation) + 32 (**symptoms management**) + unclassified + 17.1 **(no midazolam**) => 9 uncertain CDSUD

25 (yes) + 17 (yes) + 17.3 (vigil sedation) +32 (**symptoms management**) + unclassified + 17.1 (**midazolam**) => 20 probable CDSUD

25 (yes) + 17 (yes) + 17.3 (vigil sedation) + 32 (**decision of no treatment/stop life-sustaining treatment**) + 17.1 (no midazolam) => 1 uncertain CDSUD

25 (yes) + 17 (yes) + 17.3 (vigil sedation) + 32 (**decision of no treatment/stop life-sustaining treatment**) **+** 17.1 (**midazolam**) **=> 4** probable CDSUD in connexion with comments

25 (yes) + 17 (yes) + 17.3 (vigil sedation)**+ 32 (CDSUD**) =19 very likely to be CDSUD

Then continued 17.3 equation C2

**25 (yes) +17 (yes) + 17.3 (empty) = >** 3: 1 refused by the family, 1 refractory cardiorespiratory arrest and 1 already very likely to be CDSUD

- **Equation D:**

25 (yes) => 151

25 (yes) +17 (empty) => 4 including 2 certain CDSUD

+32 (empty or decision of no treatment/stop life sustaining treatment) with 17.1 (no midazolam and no morphine) => 2 unlikely to be CDSUD

**+17 (no)** => 7: 2 certain CDSUD, 1 certain CDSUD because they ticked CDSUD + euthanasia in question 32, 1 classed as certain CDSUD because they ticked CDSUD in question 32, 1 classed as very likely to be CDSUD because sedation was carried out urgently in the presence of respiratory signs, the others classified as uncertain CDSUD.

**Results of equations A+B+C+D => 44 certain CDSUD + 55 very likely CDSUD + 36 probable CDSUD + 13 uncertain CDSUD + 5 unlikely CDSUD + 11 unclassified**

- **Equation of 11 unclassified**

25 (no o empty) **+ 32 (yes)** => 3 unclassified: 1 did not answer question 25 so classified as certain CDSUD, 1 sedation for terminal respiratory distress classified as certain CDSUD, 1 uncertain CDSUD because ticked no to question 25 and yes to question 32.

25 (no o empty) + 32 ticked multiple answers to question 32 including CDSUD = 3 unclassified were classified as uncertain CDSUD

25 who answered to question 25.1 or 25.3 and put no or empty to question 25 = 5 unclassified => classified as unlikely CDSUD

**Results of equations A+B+C+D+11 unclassified => 46 certain CDSUD + 55 very likely CDSUD + 36 probable CDSUD + 17 uncertain CDSUD + 10 unlikely CDSUD**
