# Supplementary material for: Deep and continuous sedation until death in the French overseas departments
Source: PLoS One. 2025 Dec 5;20(12):e0337969. doi: 10.1371/journal.pone.0337969 (PMC12680175; doi:10.1371/journal.pone.0337969)
Supplement: S4 File — (DOCX) [file pone.0337969.s004.docx]

Supporting material 4: physician’s ranking of questionnaires

We isolated the 164 questionnaires that had “yes” answers to question 25 and/or “CDSUD” answers to question 32. Then the two physicians (AE and HB) classified it into three categories: certain, uncertain or unlikely sedation. The initial concordance rate between the two reviewers was 76% (Table: Lecture 1).There were forty classification discrepancies after their first reading. After discussion of the cases, the discrepancies were reduced to thirteen (Table: Lecture 2), agreement increased to 92%.

A collegial analysis by videoconference, which also included S.P. (co-author of this work), demographer, led to a consensus on all cases: 128 certain CDSUD, 20 uncertain CDSUD and 16 unlikely CDSUD (Table: Result of collegial analysis).

During this adjudication process, some cases initially classified as declarations of “intentional euthanasia” were reclassified as probable or uncertain deep continuous sedation until death, based on answers to other relevant questions. Conversely, a few cases initially labeled as certain sedation but showing some ambiguities were reclassified as probable when the review of responses suggested overall consistency with deep continuous sedation, except for the specific treatments mentioned.

| LECTURE 1 | | | |
| --- | --- | --- | --- |
| 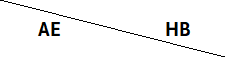 | probable CDSUD | uncertain CDSUD | unlikely CDSUD |
| Probable CDSUD | 115 | 9 | 4 |
| Uncertain CDSUD | 10 | 5 | 2 |
| Unlikely CDSUD | 12 | 3 | 4 |

| LECTURE 2 | | | |
| --- | --- | --- | --- |
| 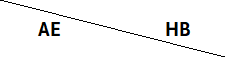 | probable CDSUD | uncertain CDSUD | unlikely CDSUD |
| Probable CDSUD | 121 | 2 | 5 |
| Uncertain CDSUD | 4 | 15 | 1 |
| Unlikely CDSUD | 1 | 0 | 15 |

| Result of collegial analysis | |
| --- | --- |
| CDSUD | |
| Probable CDSUD | 128 |
| No CDSUD | |
| Uncertain CDSUD | 20 |
| Unlikely CDSUD | 16 |
| Total | 164 |
