# Supplementary material for: Deep and continuous sedation until death in the French overseas departments
Source: PLoS One. 2025 Dec 5;20(12):e0337969. doi: 10.1371/journal.pone.0337969 (PMC12680175; doi:10.1371/journal.pone.0337969)

Supporting material 5: logistic regression

The logistic regressions aim to get a better understanding of the profile of deaths after CDSUD compared to deaths with no CDSUD.

As regressions techniques remove missing values of all variables of the model, the size of the population we based the regression is slightly different of the descriptive statistics (100.95 deaths with CDSUD and 783.68 deaths with no CDSUD).

In the regression model, we included the variables concerning the physician, the deceased person and a few variables about the end of life (cause of death, cognitive impairment, artificial hydration and nutrition, pain and some variables relative to opinions of the physician about the end of life).

CDSUD is associate with a physician that is not a general practitioner, deceased person is under 80 and the younger there are the most likely they will receive CDSUD compared to persons dead at 80 years and over. Artificial nutrition stopped a few hours before death is associated with CDSUD compared to those with artificial nutrition. The cases where the doctor does not wish to comment on the question of how they consider the end of life to have been, are negatively associated to CDSUD.

**Figure S5: logistic regression – CDSUD vs non CDSUD**


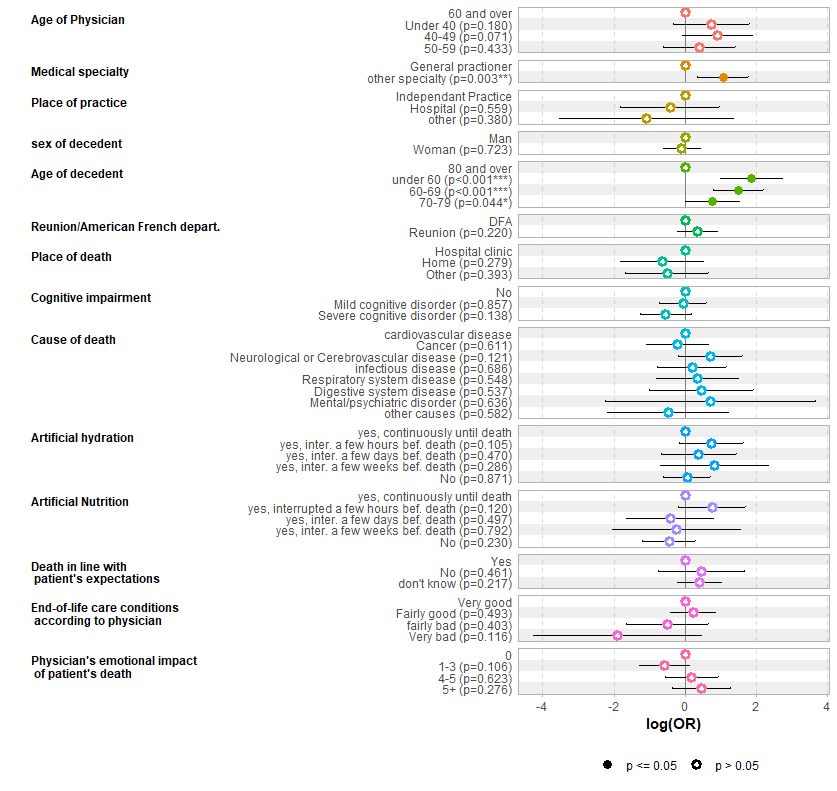

Supplement: S5 File — (DOCX) [file pone.0337969.s005.docx]
