# Supplementary material for: Deep and continuous sedation until death in the French overseas departments
Source: PLoS One. 2025 Dec 5;20(12):e0337969. doi: 10.1371/journal.pone.0337969 (PMC12680175; doi:10.1371/journal.pone.0337969)
Supplement: S6 File — (DOCX) [file pone.0337969.s006.docx]

Supporting material 6: Physician's perceptions of end-of-life and impact of sedative practices

Figure S6: Physician's perceptions of end-of-life and impact of sedative practices


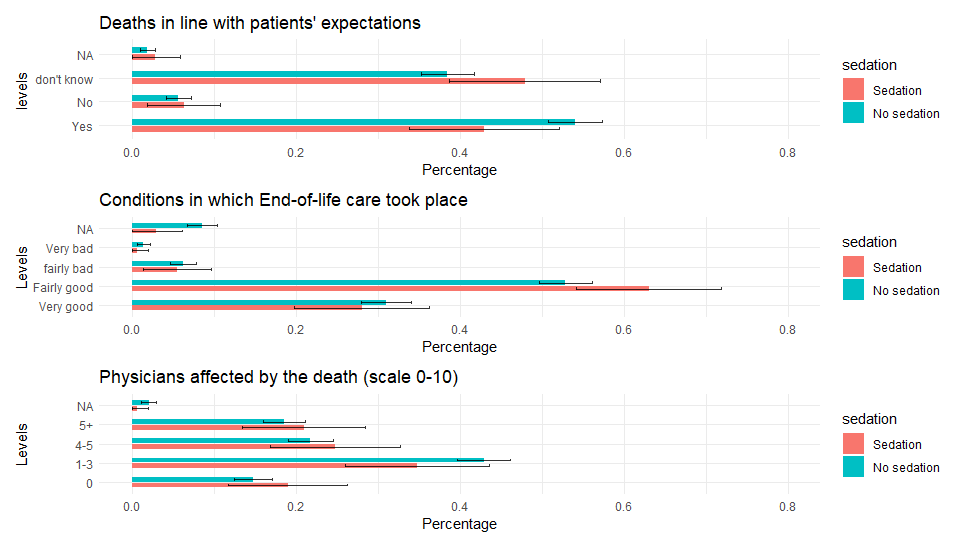


Legend:

NA : Not available

Red: CDSUD and Blue: no CDSUD
