# Supplementary material for: Deep and continuous sedation until death in the French overseas departments
Source: PLoS One. 2025 Dec 5;20(12):e0337969. doi: 10.1371/journal.pone.0337969 (PMC12680175; doi:10.1371/journal.pone.0337969)
Supplement: Attachment — Submitted filename: Letter_editor_revisionsv3.docx [file pone.0337969.s008.docx]

**PlosOne “How is deep and continuous sedation until death practiced in the French overseas departments?”**

Dear Editor and reviewers,

We thank you for your thorough reading of our paper and your suggestions that helped us to improve our manuscript.

You will find below, all our responses to your questions and suggestions.

Followed your request about the role of Funders, can you, please, change the online mention to: “The funders had no role in the study design, data collection and analysis, decision to publish, or preparation of the manuscript”.

We checked the style requirements and hope not having missed any.

Yours sincerely,

The authors

Editor

When submitting your revision, we need you to address these additional requirements.

1. Please ensure that your manuscript meets PLOS ONE's style requirements, including those for file naming. The PLOS ONE style templates can be found at

<https://journals.plos.org/plosone/s/file?id=wjVg/PLOSOne_formatting_sample_main_body.pdf> and

[https://journals.plos.org/plosone/s/file?id=ba62/PLOSOne_formatting_sample_title_authors_affiliations.pdf](https://track.editorialmanager.com/CL0/https:%2F%2Fjournals.plos.org%2Fplosone%2Fs%2Ffile%3Fid=ba62%2FPLOSOne_formatting_sample_title_authors_affiliations.pdf/1/010f0199c1b10c7d-550e5412-e83b-40ff-ada9-b0e6756cc338-000000/j95B_s0wNL2rjNRN8txRCGdea4b3Qqplif3IqmXzvPg=232)

We have followed the PLOS ONE’s style requirement, Compared to the previous version:

- titles has been transformed in sentence case and only the first letter in capital

- captions, in-text citations of figures, tables, supplementary material has been checked

- in text citations changed to square brackets

- affiliations checked

2. Please provide additional details regarding participant consent. In the ethics statement in the Methods and online submission information, please ensure that you have specified what type you obtained (for instance, written or verbal, and if verbal, how it was documented and witnessed). If your study included minors, state whether you obtained consent from parents or guardians. If the need for consent was waived by the ethics committee, please include this information.

Once you have amended this/these statement(s) in the Methods section of the manuscript, please add the same text to the “Ethics Statement” field of the submission form (via “Edit Submission”).

For additional information about PLOS ONE ethical requirements for human subjects research, please refer to [http://journals.plos.org/plosone/s/submission-guidelines#loc-human-subjects-research](http://track.editorialmanager.com/CL0/http:%2F%2Fjournals.plos.org%2Fplosone%2Fs%2Fsubmission-guidelines%23loc-human-subjects-research/1/010f0199c1b10c7d-550e5412-e83b-40ff-ada9-b0e6756cc338-000000/lXsuQvQq67ttZYuLq7-KpuNiqJKaWrNklGm2CN1zUic=232).

For your information, the project went through a double layer of ethics. One is The Comité d’Expertise pour les Recherches, les Études et les Évaluations dans le domaine de la Santé [National Expert Committee for Research, Studies and Evaluations in the Field of Health], and the second is the Commission nationale de l’informatique et des libertés [CNIL-National committee on digital and liberty – liberty act.

On one of the Cnil flyer, research on dead persons is mentioned as followed (our translation): “With regard to the processing of data relating to deceased persons, provided that the professional participating in the research is aware of the vital status of the person concerned and that the latter did not object to this in writing during their lifetime, personal data relating to them may be processed for research purposes”. (Methodology reference MR004)

We altered the sentence both in the methods/ethics statement section and in the declaration /ethical standards section to make clearer that the need for consent was waived by a national ethics committee.

As follows:

The Comité d’Expertise pour les Recherches, les Études et les Évaluations dans le domaine de la Santé [National Expert Committee for Research, Studies and Evaluations in the Field of Health], approved the methodology used, waived the need for consent and waived the requirement of medical confidentiality for research purposes (approval in March 2018)

3. Thank you for stating the following financial disclosure:

 [SP received grants to support the research by the Caisse nationale de solidarité pour l’autonomie (CNSA), as part of the call for projects launched by IReSP (project IReSP-17-Hand8-16) ; by the Fondation de France as part of the call for project “Soigner, soulager, accompagner (2017)”. The Institut national d’études démographiques (INED) was involved in the data collection through its Survey department.

<https://iresp.net/>

<https://www.fondationdefrance.org/fr/>

<https://www.ined.fr>

No funding bodies was involved in the contents of this paper.].

Please state what role the funders took in the study.  If the funders had no role, please state: "The funders had no role in study design, data collection and analysis, decision to publish, or preparation of the manuscript."

We have included this statement on the online financial statement box

If this statement is not correct you must amend it as needed.

Please include this amended Role of Funder statement in your cover letter; we will change the online submission form on your behalf.

We have included this statement on the cover letter

4. In the online submission form, you indicated that [The survey dataset analysed in the current study is not publicly available yet due to its embargo. Data can be accessible under reasonable request upon the corresponding author after approval of a proposal and with a signed access agreement.].

All PLOS journals now require all data underlying the findings described in their manuscript to be freely available to other researchers, either 1. In a public repository, 2. Within the manuscript itself, or 3. Uploaded as supplementary information.

This policy applies to all data except where public deposition would breach compliance with the protocol approved by your research ethics board. If your data cannot be made publicly available for ethical or legal reasons (e.g., public availability would compromise patient privacy), please explain your reasons on resubmission and your exemption request will be escalated for approval.

We understand your point and Ined as a research institute and data provider is sharing the principle of “open science” and open data as much as possible. In that particular case, some restrictions applies for legal reasons.

We altered the online submission form with the following statement:

Data cannot be shared freely because the department of residence/death can be identified from our dataset and that is considered identifying information by the French liberty act (CNIL). Data can be accessed by researchers affiliated to a research/educational institution, upon submission of a research project through the data progedo plateform (<https://data.progedo.fr/>). The European General Data Protection Regulation (GDPR) applies.

5. If the reviewer comments include a recommendation to cite specific previously published works, please review and evaluate these publications to determine whether they are relevant and should be cited. There is no requirement to cite these works unless the editor has indicated otherwise.

Additional Editor Comments (if provided):[Note: HTML markup is below. Please do not edit.]

Reviewers' comments:

Reviewer's Responses to Questions

**Comments to the Author**

1. Is the manuscript technically sound, and do the data support the conclusions?

The manuscript must describe a technically sound piece of scientific research with data that supports the conclusions. Experiments must have been conducted rigorously, with appropriate controls, replication, and sample sizes. The conclusions must be drawn appropriately based on the data presented.

Reviewer #1: Yes

Reviewer #2: Yes

2. Has the statistical analysis been performed appropriately and rigorously?

Reviewer #1: Yes

Reviewer #2: Yes

3. Have the authors made all data underlying the findings in their manuscript fully available?

The [PLOS Data policy](http://track.editorialmanager.com/CL0/http:%2F%2Fwww.plosone.org%2Fstatic%2Fpolicies.action%23sharing/1/010f0199c1b10c7d-550e5412-e83b-40ff-ada9-b0e6756cc338-000000/6oPpjXWi1C0MUZ56ey21xASy5foZowYJFeTrx-AS4RE=232) requires authors to make all data underlying the findings described in their manuscript fully available without restriction, with rare exception (please refer to the Data Availability Statement in the manuscript PDF file). The data should be provided as part of the manuscript or its supporting information, or deposited to a public repository. For example, in addition to summary statistics, the data points behind means, medians and variance measures should be available. If there are restrictions on publicly sharing data—e.g. participant privacy or use of data from a third party—those must be specified.

Reviewer #1: Yes

Reviewer #2: Yes

4. Is the manuscript presented in an intelligible fashion and written in standard English?

PLOS ONE does not copyedit accepted manuscripts, so the language in submitted articles must be clear, correct, and unambiguous. Any typographical or grammatical errors should be corrected at revision, so please note any specific errors here.

Reviewer #1: No

Reviewer #2: Yes

The manuscript has been revised by a native English-speaking language professional editor

5. Review Comments to the Author

Please use the space provided to explain your answers to the questions above. You may also include additional comments for the author, including concerns about dual publication, research ethics, or publication ethics. (Please upload your review as an attachment if it exceeds 20,000 characters)

Reviewer #1: This study addresses continuous deep sedation until death (CDSUD) as practiced in the French overseas departments. The methodology consisted of surveying physicians who had completed the death certificates and comparing those who performed CDSUD with those who did not. The article is well written, clear, and concise. I have only two comments:

We thank you for assessing our paper and for your suggestions that we took into account in the revision of our manuscript

R1.1-The tables presenting only the weighted frequencies and weighted percentages are not straightforward to interpret. It would be more informative for the reader if a table with three columns were provided: the first including the unweighted counts and percentages (n), the second the weighted frequencies and weighted percentages (n ), and the third the p-value.

We changed the tables in order to comply with your proposal and displayed in tables both the unweighted and weighted frequencies and percentages. Supplementary file 7 with unweighted data was removed, as it is no longer useful.

R1.2-Considering the importance of the topic and the data collected in the overseas territories, it is regrettable that practitioners working in these territories were not involved in this publication. I am not convinced that physicians (VG and AE) from Nantes (mainland France), authors of this article, would have appreciated researchers from the overseas departments analyzing and using data from their hospital without being associated with the research. It even appears that physicians from La Réunion were approached to discuss the results. There are established teams and palliative care units in these territories, as well as (future) academics seeking to specialize in palliative care; excluding them from the study borders on disregard.

We understand the concerns you have raised regarding the principle, and we believe that the opportunity to get involved in studies should be given to locals. This research project and its protocol were adapted to overseas regions from those initially developed for mainland France, after a request from local health authorities. Local physicians were involved from preliminary discussions to first results presentations, without any of them showing interest in joining the research team, despite invitations to do so.

Reviewer #2: Overall assessment

This manuscript offers a valuable and original contribution by investigating continuous deep sedation until death (CDSUD) in the French overseas departments. The study addresses an important knowledge gap and provides new insights into the implementation of a unique legal framework. The design is thoughtful, and the results are of international interest.

That said, certain aspects require clarification or expansion before the paper can reach its full potential. These issues are not fundamental flaws, but they do limit the current strength of the conclusions.

We thank you for your thorough review of our paper and we have done our best to respond to all your comments/suggestions.

**Major comments**

*R2.1 Response rate*

The relatively low response rate (22.9%) is a key limitation. While this is common in physician surveys, some additional detail on representativeness (e.g., how respondents compared with the overall physician population in the overseas departments) would reassure readers about the reliability of prevalence estimates.

We cannot compare the responding physicians with the overall profile of physicians in these regions, since not all physicians are certifying doctors. Furthermore, the profile of certifying physicians is not a known statistic.

Additionally, while the respondents are physicians, the sample is actually a sample of deaths. The same physician may have responded for multiple deaths. If the certifying physicians were not the physician in charge of the deceased person, he/she could transfer the questionnaire to his/her colleague who was better able to respond.

However, we have clarified that we weighted the sample of responses to ensure representativeness with respect to all deaths during the period and mentioned that the weighting did not reveal any significant distortion between the distribution by sex, age, place of death, data collection period and overseas department.

We added the following paragraph in the methods section to replace the initial statements, in the statistical analysis section related to weighting process.

**Representativeness of the responses**

To ensure the representativeness of deaths, data were adjusted and standardised based on sex, age, place of death, data collection period, and overseas Department.

The differences between the responses and the total number of deaths over the period were minimal in terms of gender, age group and place of death. Unsurprisingly, they are slightly higher for later collection periods, probably because we were interviewing some of the same doctors. The main difference was between territories, with a higher response rate for La Réunion (23%) than for the other territories (15.4-19.7%). We used for the results the weighted frequencies and percentages to give a better representation of our initial population of deaths.

*R2.2-Case classification*

The adjudication process for CDSUD cases is innovative, but it introduces subjectivity. A clearer description of how disagreements were handled, or a basic measure of agreement between reviewers, would strengthen the credibility of this approach.

Thank you for your comment. We made the modifications in Supporting Material 4. We highlighted the agreement rate between evaluators and noted some elements to illustrate the changes.*R2.3-Clinical information*

Because the study relies on death certificates, clinical details such as disease stage, comorbidities, and symptom burden are missing. Explicit acknowledgment of this limitation is needed, as it affects interpretation of appropriateness of CDSUD.

We added this information in the limitation section of the discussion (lines 414-416)

“We did not had variables on clinical details such as disease stage, comorbidities or symptom burden which may have limited our possibility to acknowledge the appropriateness of CDSUD”.

*R2.4 - Collegial procedure*

The finding that only about one-third of cases followed the formal collegial procedure is highly relevant. The discussion would benefit from exploring possible reasons for this gap (e.g., time constraints, institutional barriers, cultural norms) and its implications for policy and practice).

We altered the conclusion to introduce this idea (line 439-441)

“Discussion of the decision was frequent, although not always organised and formalised as a formal collegial procedure, indicating an area for improvement”.

*R2.5-Cultural context*

The overseas departments have distinctive cultural and religious environments, which likely influence end-of-life practices. A more developed reflection on this dimension would enrich the discussion and make the findings more interpretable for international readers.

We agree that cultural and religious contexts may influence end-of-life practices. We tried to addressed them in the manuscript, both in the contextual section (lines 84–85) and further discussed in the Discussion (line 337)."

**Minor comments**

R2.6 -Be consistent in distinguishing weighted vs. unweighted data throughout the text and tables.

The tables was reformatted as suggested. In the text, we use only weighted frequencies and percentage

R2.7- Consider adding a simple figure (e.g., prevalence of CDSUD by age or place of death) to complement the detailed tables.

We have considered adding the figures proposed. To be consistent with the tables, we have to use column percentages for the graphs and we have considered that stacked histogram were the correct graphs.

We are proposing to add these two diagrams as one figure.

R2.8-Define French-specific terms (e.g., collegial procedure) to ensure accessibility for international readers.

Following your advice, we added a definition for advance directives (line 59-61), and altered the one on collegial procedure line 64-65.

R2.9 The introduction and discussion could emphasize more clearly how this study builds upon and differs from previous European and international studies of sedation.

We believe this point was already addressed in the Introduction and in the Discussion. In the Introduction, we highlighted the limited availability of international data on CDSUD, the small sample sizes of previous French studies, and the existing gaps regarding patient characteristics, indications, and physician experiences, including emotional and ethical aspects. In the Discussion, we further contextualised our findings within French and European literature, emphasizing differences in prevalence, legal frameworks, and organizational contexts, as well as the specific features of overseas France.

We would be happy to follow more precise suggestions from reviewers to make our points clearer

6. PLOS authors have the option to publish the peer review history of their article ([what does this mean?](https://track.editorialmanager.com/CL0/https:%2F%2Fjournals.plos.org%2Fplosone%2Fs%2Feditorial-and-peer-review-process%23loc-peer-review-history/1/010f0199c1b10c7d-550e5412-e83b-40ff-ada9-b0e6756cc338-000000/iNIG_esP3_qADJG7_6lKfs9lga6ykamD5OermAcCk-k=232)). If published, this will include your full peer review and any attached files.

If you choose “no”, your identity will remain anonymous but your review may still be made public.

**Do you want your identity to be public for this peer review?** For information about this choice, including consent withdrawal, please see our [Privacy Policy](https://track.editorialmanager.com/CL0/https:%2F%2Fwww.plos.org%2Fprivacy-policy/1/010f0199c1b10c7d-550e5412-e83b-40ff-ada9-b0e6756cc338-000000/ENuFWSwxu1ttrz_UX6CWpt2rcA5Ae8n7ZBkENsJb_0o=232).

Reviewer #1: **Yes:** Denis Boucaud-Maitre

Reviewer #2: No

[NOTE: If reviewer comments were submitted as an attachment file, they will be attached to this email and accessible via the submission site. Please log into your account, locate the manuscript record, and check for the action link "View Attachments". If this link does not appear, there are no attachment files.]

While revising your submission, please upload your figure files to the Preflight Analysis and Conversion Engine (PACE) digital diagnostic tool, <https://pacev2.apexcovantage.com/>. PACE helps ensure that figures meet PLOS requirements. To use PACE, you must first register as a user. Registration is free. Then, login and navigate to the UPLOAD tab, where you will find detailed instructions on how to use the tool. If you encounter any issues or have any questions when using PACE, please email PLOS at [figures@plos.org](mailto:figures@plos.org). Please note that Supporting Information files do not need this step.

 We have used the PACE tool to ensure that our figures meet PLOS requirements.

*In compliance with data protection regulations, you may request that we remove your personal registration details at any time.* [*(Remove my information/details)*](https://track.editorialmanager.com/CL0/https:%2F%2Fwww.editorialmanager.com%2Fpone%2Flogin.asp%3Fa=r/1/010f0199c1b10c7d-550e5412-e83b-40ff-ada9-b0e6756cc338-000000/ZRw-cWXi5CSKar2BFlk679Wo4w9LY_zOmSjZCyMgOZk=232)*. Please contact the publication office if you have any questions.*
