# Supplementary material for: Deep and continuous sedation until death in the French overseas departments
Source: PLoS One. 2025 Dec 5;20(12):e0337969. doi: 10.1371/journal.pone.0337969 (PMC12680175; doi:10.1371/journal.pone.0337969)
Supplement: Attachment — Submitted filename: Letter_editor_2ndround_Revision.docx [file pone.0337969.s009.docx]

Dear Editor,

Please find below our detailed responses to the comments raised by Reviewer 1. We have done our utmost to address each point. Regarding the suggestion to differentiate the results by territory, while we recognize its relevance, we were unfortunately unable to implement it due to data limitations and ethical constraints.

We hope that these revisions meet the reviewer’s expectations and look forward to your feedback.

Best regards,

The authors.

Dear Reviewer,

Reviewer #1:

We thank you for your review and the time you have dedicated to evaluating our manuscript. We have done our best to address all your comments and hope that the revisions now meet your expectations.

1.The authors have only partially addressed my previous comment regarding their limited familiarity with the French overseas territories (DOM) and the absence of collaboration with local practitioners.

We fully acknowledge the importance of involving local practitioners in research conducted in their territories, and we appreciate the opportunity to clarify our approach in response to your concerns.

The original protocol for this study was designed for mainland France, based on established methodologies used in other European countries. Following a request from one of the Regional Health Agencies of the French overseas territories, we adapted and extended the survey to these regions. Before initiating the study, we engaged in preliminary discussions with local physicians in one of the territories to assess the relevance and feasibility of the project. After data collection, we also presented our initial findings to local practitioners during dedicated meetings explicitly inviting them to participate in the research team if they wished to do so. While these exchanges did not result in any formal collaboration, we remain committed to fostering partnerships with local teams in future research.

2. For example, they state that cancers are the leading cause of mortality (lines 85–86), which does not reflect the situation in the French West Indies, where cardiovascular diseases remain the main cause of death.

We have checked this information. We wrote this sentence after analysing data from the centre of epidemiology of causes of deaths (CepiDc-Inserm) - the open data portal-. The table below gives the number of deaths by neoplasms and disease of circulatory system and the percentage per year and the figure gives the results by overseas departments.

During the last few years, the two main causes of deaths are neoplasms and diseases of circulatory systems, but neoplasms are now slightly above. To take into account your concern, we have altered the text:

Line 86: The French overseas departments operate under the same legal framework as mainland France and exhibit a similar distribution of causes of death. Over recent years, cancer has emerged as the leading cause of mortality, although cardiovascular disease persists at a high level—indeed higher than in mainland France [15].

Table 1 gives the number of deaths and percentage 2010-2013 of Guadeloupe, Martinique, French Guiana and La Réunion (both sexes, all ages)

Source: <https://opendata-cepidc.inserm.fr/>

[Fouillet A, Aubineau Y, Godet F, Costemalle V, Coudin É., Grandes causes de mortalité en France en 2023 et tendances récentes, Bull Épidémiol Hebd., 2025;(13):218-243.](http://beh.santepubliquefrance.fr/beh/2025/13/2025_13_1.html)

[Godet F, Costemalle V, Aubineau Y, Fouillet A, Coudin É., Causes de décès en France en 2023 : des disparités territoriales, DREES, Études et Résultats n°1342](https://drees.solidarites-sante.gouv.fr/publications-communique-de-presse/250724_ER_mortalite-en-france-en-2023)

Figure R1: Number of deaths by neoplasms (blue) and disease of circulatory system (orange) by department.

Guadeloupe

|  | 971 2. Tumeurs | 971 7. Maladies de l’appareil circulatoire |
| --- | --- | --- |
| 2010 | 726 | 780 |
| 2011 | 679 | 741 |
| 2012 | 704 | 701 |
| 2013 | 695 | 711 |
| 2014 | 714 | 780 |
| 2015 | 710 | 637 |
| 2016 | 767 | 761 |
| 2017 | 685 | 652 |
| 2018 | 814 | 689 |
| 2019 | 870 | 788 |
| 2020 | 837 | 774 |
| 2021 | 841 | 794 |
| 2022 | 904 | 786 |
| 2023 | 889 | 755 |

Martinique

|  | 2. Tumeurs | 7. Maladies de l’appareil circulatoire |
| --- | --- | --- |
| 2010 | 779 | 734 |
| 2011 | 765 | 697 |
| 2012 | 473 | 441 |
| 2013 | 823 | 700 |
| 2014 | 826 | 819 |
| 2015 | 806 | 749 |
| 2016 | 845 | 818 |
| 2017 | 898 | 778 |
| 2018 | 831 | 747 |
| 2019 | 905 | 834 |
| 2020 | 916 | 773 |
| 2021 | 907 | 847 |
| 2022 | 831 | 791 |
| 2023 | 889 | 721 |

French Guiana

|  | 2. Tumeurs | 7. Maladies de l’appareil circulatoire |
| --- | --- | --- |
| 2010 | 131 | 145 |
| 2011 | 137 | 132 |
| 2012 | 147 | 141 |
| 2013 | 130 | 133 |
| 2014 | 154 | 144 |
| 2015 | 164 | 173 |
| 2016 | 192 | 195 |
| 2017 | 191 | 221 |
| 2018 | 183 | 177 |
| 2019 | 206 | 207 |
| 2020 | 206 | 156 |
| 2021 | 199 | 199 |
| 2022 | 216 | 219 |
| 2023 | 229 | 216 |

La Réunion

|  | 2. Tumeurs | 7. Maladies de l’appareil circulatoire |
| --- | --- | --- |
| 2010 | 1006 | 1227 |
| 2011 | 982 | 1112 |
| 2012 | 1105 | 1078 |
| 2013 | 1001 | 1053 |
| 2014 | 1218 | 1019 |
| 2015 | 1151 | 1078 |
| 2016 | 1228 | 1173 |
| 2017 | 1213 | 1093 |
| 2018 | 1211 | 1163 |
| 2019 | 1214 | 1230 |
| 2020 | 1346 | 1178 |
| 2021 | 1285 | 1174 |
| 2022 | 1252 | 1239 |
| 2023 | 1439 | 1193 |

3. Moreover, the French West Indies and Réunion have distinct clinical profiles and healthcare structures. Conducting additional analyses to differentiate findings between these two territories would strengthen the study and enhance its relevance for clinicians working in these regions.

We fully agree that the French West Indies and Réunion exhibit distinct clinical profiles and healthcare structures, despite sharing the same regulatory framework as mainland France. In our study, we already highlighted key structural differences, such as the higher proportion of deaths occurring at home, largely due to the lack of nursing home facilities in these territories.

In the discussion, we noted the significant differences in prevalence between the overseas departments, while exercising caution in interpreting these findings given the overall number of cases. Specifically, we reported a significantly higher prevalence of CDSUD in Réunion compared to the French West Indies and French Guiana. However, the limited number of CDSUD cases precluded a robust comparison of deceased patient or physician profiles by department, even when pooling data from the West Indies.

In the limitations section, we acknowledge that further territory-specific analyses—which would have been highly relevant for local practitioners—could not be performed due to data constraints. Unfortunately, the sample size did not allow for more detailed comparisons, despite the clear clinical interest of such an approach.

We have mentioned in the discussion that there were differences according to departments to emphasize this result.

Line 316: Although the prevalence rates differ significantly among the overseas departments, the overall number of cases warrants caution in interpreting these findings as indicative of differences in clinical practice.

And in the limits section, we acknowledge that we could not do more that we have done due to data limitations.

Line 400: While differences in prevalence between territories may exist, the limited number of cases precluded a territory-specific analysis.
